# IFNγ-Stat1 axis drives aging-associated loss of intestinal tissue homeostasis and regeneration

**DOI:** 10.1038/s41467-023-41683-y

**Published:** 2023-09-30

**Authors:** Omid Omrani, Anna Krepelova, Seyed Mohammad Mahdi Rasa, Dovydas Sirvinskas, Jing Lu, Francesco Annunziata, George Garside, Seerat Bajwa, Susanne Reinhardt, Lisa Adam, Sandra Käppel, Nadia Ducano, Daniela Donna, Alessandro Ori, Salvatore Oliviero, Karl Lenhard Rudolph, Francesco Neri

**Affiliations:** 1 Leibniz Institute on Aging – Fritz Lipmann Institute (FLI), Jena, Germany; 2 Department of Life Sciences and Systems Biology, University of Turin, Torino, Italy; 3 Molecular Biotechnology Center, University of Turin, Torino, Italy; 4 Dresden-concept Genome Center, c/o Center for Regenerative Therapies Dresden (CRTD), Dresden, Germany

**Keywords:** Endoderm, Gastrointestinal models, Intestinal stem cells

## Abstract

The influence of aging on intestinal stem cells and their niche can explain underlying causes for perturbation in their function observed during aging. Molecular mechanisms for such a decrease in the functionality of intestinal stem cells during aging remain largely undetermined. Using transcriptome-wide approaches, our study demonstrates that aging intestinal stem cells strongly upregulate antigen presenting pathway genes and over-express secretory lineage marker genes resulting in lineage skewed differentiation into the secretory lineage and strong upregulation of MHC class II antigens in the aged intestinal epithelium. Mechanistically, we identified an increase in proinflammatory cells in the *lamina propria* as the main source of elevated interferon gamma (IFNγ) in the aged intestine, that leads to the induction of Stat1 activity in intestinal stem cells thus priming the aberrant differentiation and elevated antigen presentation in epithelial cells. Of note, systemic inhibition of IFNγ-signaling completely reverses these aging phenotypes and reinstalls regenerative capacity of the aged intestinal epithelium.

## Abstract (web summary)

Omrani, Krepelova *et al*. report that aging-induced proinflammatory IFNγ/Stat1 signalling primes intestinal stem cells to a secretory fate and to antigen presenting cells impairing the regenerative capacity of the aging gut epithelium.

## Introduction

Aging is the most prevalent risk factor for cardiovascular disease and cancer, which affects the homeostasis of all tissues in the body^1,2^. Intestinal epithelium is one of the most proliferating tissues in the body and has a variety of different functional roles, including nutrient absorption and barrier function. Intestinal stem cells (ISCs), located at the base of intestinal crypts, fuel this highly dynamic tissue by continuous daily divisions and differentiating towards absorptive or secretory cells^3^. ISCs show a functional decline in old organisms. Aged ISCs have less proliferative and regenerative capacity, and are accompanied by an increased number of differentiated cells in the tissue^4–11^. The molecular mechanisms for such a decrease in the functionality of ISCs during aging remain largely unknown, even though a variety of molecular defects such as perturbation in WNT and mTOR signaling and reduction of fatty-acid oxidation have been recently reported^4–7^. The influence of aging on crypt cells and their microenvironment can further explain the underlying causes for the loss of functionality of the ISCs.

Intestinal epithelium and especially ISCs respond to signals coming from surrounding niches, such as the gut lumen and neighboring immune cells^12–14^. Aging has been demonstrated to influence these compartments, and these aging-associated alterations contribute to the inflammation process, a chronic systemic inflammatory process that is one of the hallmarks of aging^15–21^.

In the intestinal lamina propria, several types of immune cells are in close contact with intestinal epithelial cells. In homeostatic and inflammatory conditions, like infection, cytokines are secreted by different immune cells to trigger differentiation of ISCs and remodeling of the intestinal tissue to cope with environmental cues recognized by immune cells^13,22^.

However, how the homeostatic crosstalk between immune and epithelial cells is altered during aging is currently unknown. Here, by using different sequencing approaches, including scRNA-seq of intestinal crypt cells and lamina propria immune cells, we show that an aging-associated increase in inflammation primes ISCs to differentiate into secretory epithelial cells and to exhibit aberrant upregulation of antigen presentation genes. The study reveals increases in pro-inflammatory immune cells (T cells and innate lymphoid cell type 2) in the lamina propria as the main source for aging-related increase of interferon-gamma/Stat1 signaling in the intestinal epithelium. Importantly, blockage of interferon signaling reverts these aging-related changes in the intestinal epithelium and rejuvenates its regenerative capacity in response to injury.

## Results

### Overexpression of major histocompatibility complex II during aging

**Fig. 1:**
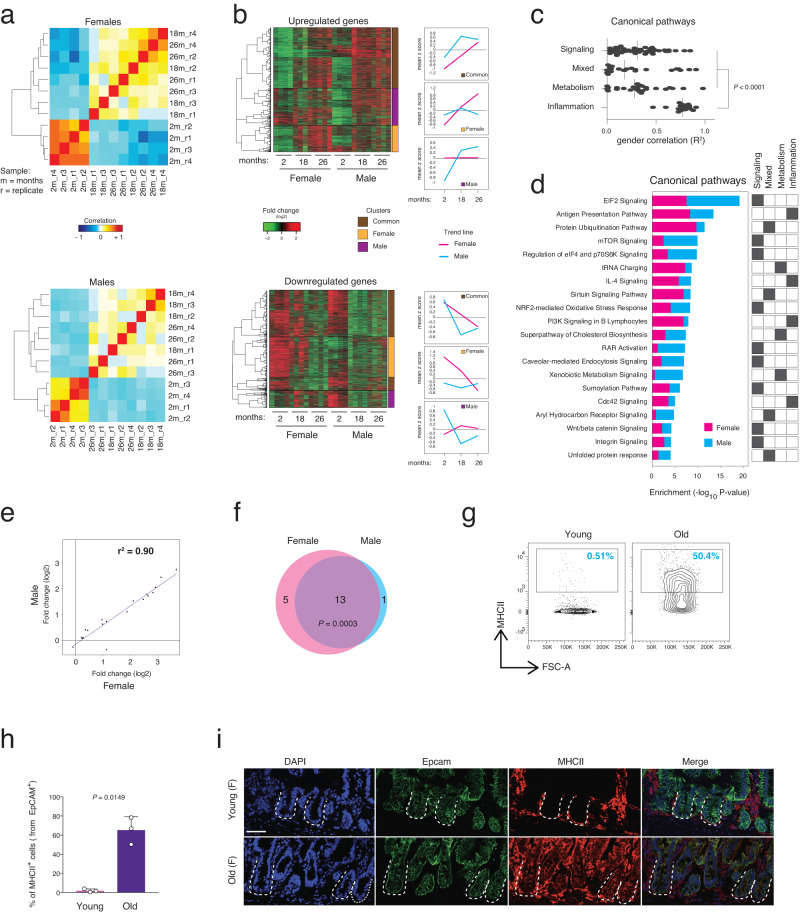
Major histocompatibility complex class II (MHCII) is strongly overexpressed during aging in whole intestinal crypts in both genders. **a** Hierarchical clustering and heatmap of the Pearson correlation of the RNA-seq datasets of mouse intestinal crypts at the indicated ages. *n* = 4 mice per group were analyzed. **b** Left panels showing hierarchical clustering and heatmap of the expression level of the differentially expressed genes (DEGs) in the same datasets as in Fig. 1a. Right panels showing line-plots of DEG regulation (mean of gene base scaled count) during aging that are divided into indicated clusters of genes. **c** Point plot of the gender correlation (*R*^2^) of the DEGs found in each gene ontology (GO) pathways divided by the indicated main GO categories. Vertical lines indicate median. *P* value was calculated by Mann-Whitney two-tail test. **d** Stacked bar chart of −log10 enrichment *p* value (Fisher two-sided exact test) in the most relevant GO pathways found dysregulated in aging. GO categories were assigned by following the clustering in Fig. S1d. **e** Scatter plot of the log2 fold change of the DEGs during aging in the antigen-presenting pathway. *r* = coefficient of determination. **f** Venn diagram of the number of the DEGs in female and male mice in antigen-presenting pathway as in Fig. 1e. *P* value was calculated by hypergeometric two-sided distribution test. High *P* value indicating a significant gene overlap. **g** Representative FACS plots showing percentages of MHCII^+^ cells in the indicated conditions. **h** The bar chart shows the percentage of MHCII^+^ cells from EpCAM^+^ cells in the indicated groups. *n* = 3 mice per group were analyzed. Error bars represent SD. *P* value was calculated by two-sided Welch’s *t* test. **i** Representative pictures of Major Histocompatibility Complex class II (MHCII-IA) stain the H2-Aa and H2-Ab1 heterodimer of the Antigen Presentation Pathway in young and aged intestinal crypts. The dotted lines indicate the crypt structure. Scale bar, 50 µm.

To characterize the aging-induced transcriptional changes in intestinal crypts, we performed total RNA-seq on freshly isolated intestinal crypts from male and female mice from 3 different age groups (young, 2 months old; old, 18 months old; geriatric, 26 months old). Transcriptional profiles of crypts from young mice clustered separately from the 2 groups of old mice (Fig. 1a). Moreover, the old samples show an increase in intragroup sample-to-sample distance indicating that aging leads to a larger transcriptional variance (Figs. 1a and S1a). We determined ~2500 significantly differentially expressed genes (DEGs) in both females and males during aging (Fig. S1b, Supplementary Data S1). Although the majority of the DEGs were gender-specific, ~30% of the DEGs were shared between female and male mice indicating the existence of a common aging signature in intestinal crypts (Fig. S1c). Hierarchical clustering of all the DEGs revealed gender-dependent and independent clusters of genes that were influenced by aging (Fig. 1b, left panels). Interestingly, females showed a continuous trend of regulation from 2 to 26 months of age for both upregulated and down-regulated common genes, while males displayed a U-shape or reverse U-shape profile, as previously reported in other tissues during aging^23,24^ (Fig. 1b, right panels). This gender-based difference in transcriptional profiles could indicate a different aging rate or mechanistic process between male and female intestinal crypts. Gene ontology (GO) analysis of the DEGs identified more than one hundred canonical pathways significantly enriched in old intestinal crypts indicating aging is indeed a complex multifactorial process. Since the database used for GO analysis contains many canonical pathways sharing the same genes and therefore providing redundant information, we performed a hierarchical cluster analysis of the top 120 significant pathways based on their gene overlap (Fig. S1d, Supplementary Data S2). We found four major categories of pathways related to (1) signaling, (2) proteostasis/stress response and epigenetics (hereafter called mixed), (3) metabolism, and (4) inflammation. Most of the identified canonical pathways in categories 1–3 have previously been shown to have a key role in extending organismal life-span (maintaining intestinal homeostasis during aging (e.g., Wnt/beta-catenin and aryl hydrocarbon receptor signaling^4,6,12^, quality control pathways in aging (e.g., protein ubiquitination, unfolded protein response^25^), and metabolic pathways during aging (e.g., Sirtuin and mTOR signaling^26^).

Interestingly, unlike other categories, pathways belonging to the inflammation category showed a higher gender correlation of their aging-dependent regulation (Fig. 1c). To investigate common altered transcriptome changes in the intestinal crypt cells during aging, we focused our attention to the most enriched inflammation pathway in the aged intestine, specifically the antigen-presenting pathway (APP) (Figs. 1d and S1e). This pathway showed strong correlation (*r*^2^ = 0.9) between the two genders, with almost all of the genes being equally regulated in both aged female and male mice (Fig. 1e, f). FACS and IF analysis of intestinal crypt cells from young and old mice confirmed the increase in the percentage of cells expressing the major histocompatibility complex class II (MHCII) proteins on their surface during aging (Fig. 1g–i). In agreement with these results, qRT-PCR confirmed the upregulation of mRNA level of genes belonging to APP in old intestinal crypts (Fig. S1f). APP has been recently demonstrated as an important player in mediating crosstalk between immune cells and ISCs in homeostasis or following intestinal perturbations^13,27,28^. Together, these findings indicate that intestinal crypts from male and female mice undergo a substantial amount of transcriptional changes during aging. Remarkably, changes in inflammation-related pathways including the antigen presentation pathway were evident in both genders, pointing towards the presence of a shared aging signature between the genders.

### Altered inflammation-related pathways and differentiation in aged ISCs

**Fig. 2:**
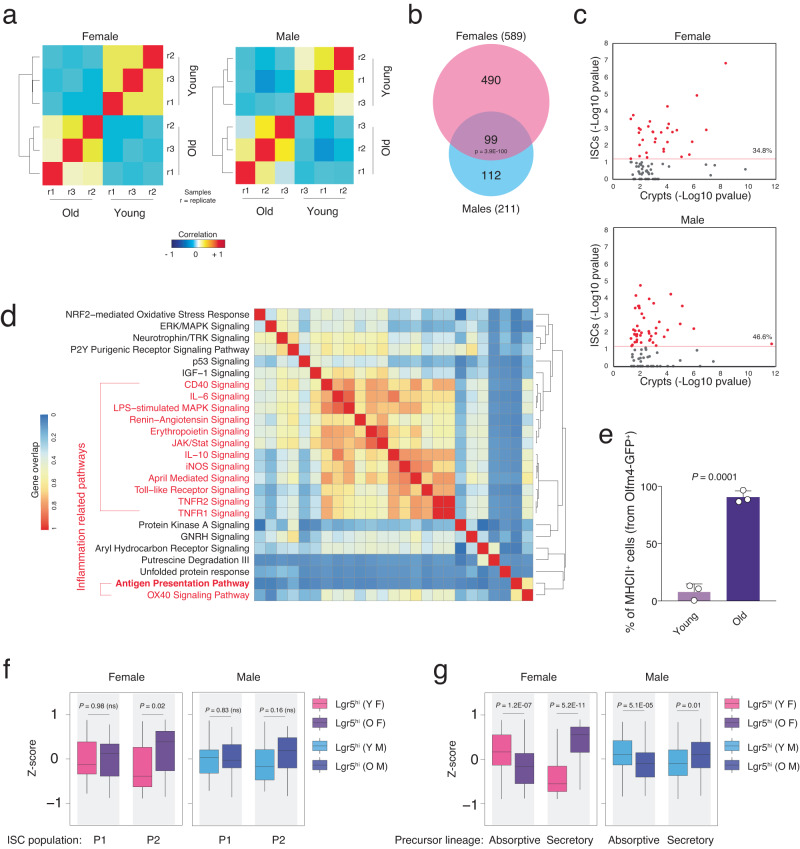
Inflammation-related pathways are enriched at the intestinal stem cell level. **a** Hierarchical clustering and heatmap of the Pearson correlation of the RNA-seq datasets of Lgr5^hi^ intestinal stem cell for indicated ages. *n* = 3 mice per group were analyzed. **b** Venn diagram of the DEGs found in the RNA-seq of the Lgr5^hi^ intestinal stem cells for the indicated genders. *P* value was calculated by two-sided hypergeometric distribution test. **c** Scatter plot of the −log10 *p* values of the enrichment score for the GO pathways found significantly enriched in the whole crypt RNA-seq. The scatter plots show the correlation between enrichment scores found in Lgr5^hi^ intestinal stem cells (Y-axis) versus whole crypts (*X* axis) RNA-seq. Percentage indicates the pathways found statistically enriched both in Lgr5^hi^ intestinal stem cells and whole crypts. **d** Hierarchical clustering and heatmap of the gene overlap correlation of the top 25 GO pathways found enriched in the Lgr5^hi^ intestinal stem cells during aging. Red-marked pathways are related to immune system signaling and inflammation. **e** The bar chart shows the percentage of MHCII^+^ cells from Olfm4^+^ intestinal stem cells in the indicated groups. *n* = 3 mice per group were analyzed. Error bars represent the SD. *P* value was calculated by two-sided Welch’s *t* test. **f**, **g** Box plot of geneset enrichment analysis of the indicated gene datasets in the Lgr5^hi^ intestinal stem cells purified from female (F) and male (M) mice, young (Y) and old (O) mice. *n* = 3 mice per group were analyzed. *P* value was calculated by two-sided Wilcoxon paired test. ns  not significant.

To study whether observed transcriptomic aging-associated changes in intestinal crypt cells are evident at the stem cell level we utilized the previously developed ISC reporter Lgr5-eGFP-CreERT2 mouse model^3^ to purify ISCs (Lgr5^hi^) of young and old mice from both genders. Similar to findings in whole intestinal crypt cells, total RNA-seq of isolated Lgr5^hi^ cells showed distinct transcriptional differences between young and old mice in both female and male animals, with significant DEG overlap among the different genders (Fig. 2a, b, Supplementary Data S1). Interestingly, gene ontology analysis of these samples revealed that almost half (34.8% in females and 46.6% in male) of the canonical pathways found enriched in crypts during aging were also significantly enriched in Lgr5^hi^ cells in old versus young mice (Fig. 2c), suggesting that many of the aging-associated transcriptional alterations found in intestinal crypts originate from changes at stem cell level. Furthermore, the commonly enriched pathways between ISCs and crypts showed a high positive correlation in the directional regulation of the genes and an over-representation of inflammation-related pathways in both genders (Fig. S2a, b). Analyzing the top 25 canonical pathways enriched in aged Lgr5^hi^ cells revealed a big cluster of inflammation-related pathways including the APP (Fig. 2d). In agreement with this finding, FACS analysis utilizing the Olfm4-eGFP-CreERT2 reporter mice^29^ revealed an increase of MHCII^+^ ISCs in old mice to almost 100% (Figs. 2e and S2c). In addition, Lgr5^hi^ ISCs exhibited an increase in pathways that have been previously reported to be dysregulated during aging but were not enriched in the analysis of whole crypts from aged versus young mice including IGF-1, p53, and calcium signaling (Fig. 2d)^30–33^. Taken together, these results indicate that aging-associated upregulation of inflammatory pathways is conserved and shared between Lgr5^hi^ cells and the differentiating/ed cells of the intestinal crypts. It has been reported that inflammation signaling and functionality of ISCs can be influenced in a cell-non-autonomous manner by neighboring immune cells^13,27,28^. On the other hand, pathways related to signaling and metabolism are rather more cell-type specific and therefore differently enriched between Lgr5^hi^ cells and whole intestinal crypt cells.

Recent study from Kim et al.^34^ has reported that Lgr5^hi^ cells are composed of two subpopulations of intestinal stem cells including naive stem cell population (P1) and a multilineage-primed subpopulation (P2). Using P1 and P2-specific gene datasets, we tested whether Lgr5^hi^ cells equally preserve the ratio of these subpopulations during aging. Interestingly, old female-derived Lgr5^hi^ cells displayed a significant enrichment of the P2 subpopulation gene dataset with a similar trend observed in old males (Fig. 2f). Geneset enrichment analysis using different datasets^35,36^ further validated this result and showed that old Lgr5^hi^ cells from both genders were significantly enriched in markers of secretory but not absorptive precursors as well as markers of fully differentiated secretory cells (except enteroendocrine cells in males) (Figs. 2g and S2d). Taken together, these data indicated a skewing at the intestinal stem cell level towards secretory lineage during aging.

### Niche-dependent alterations in the intestinal crypt cells during aging

**Fig. 3:**
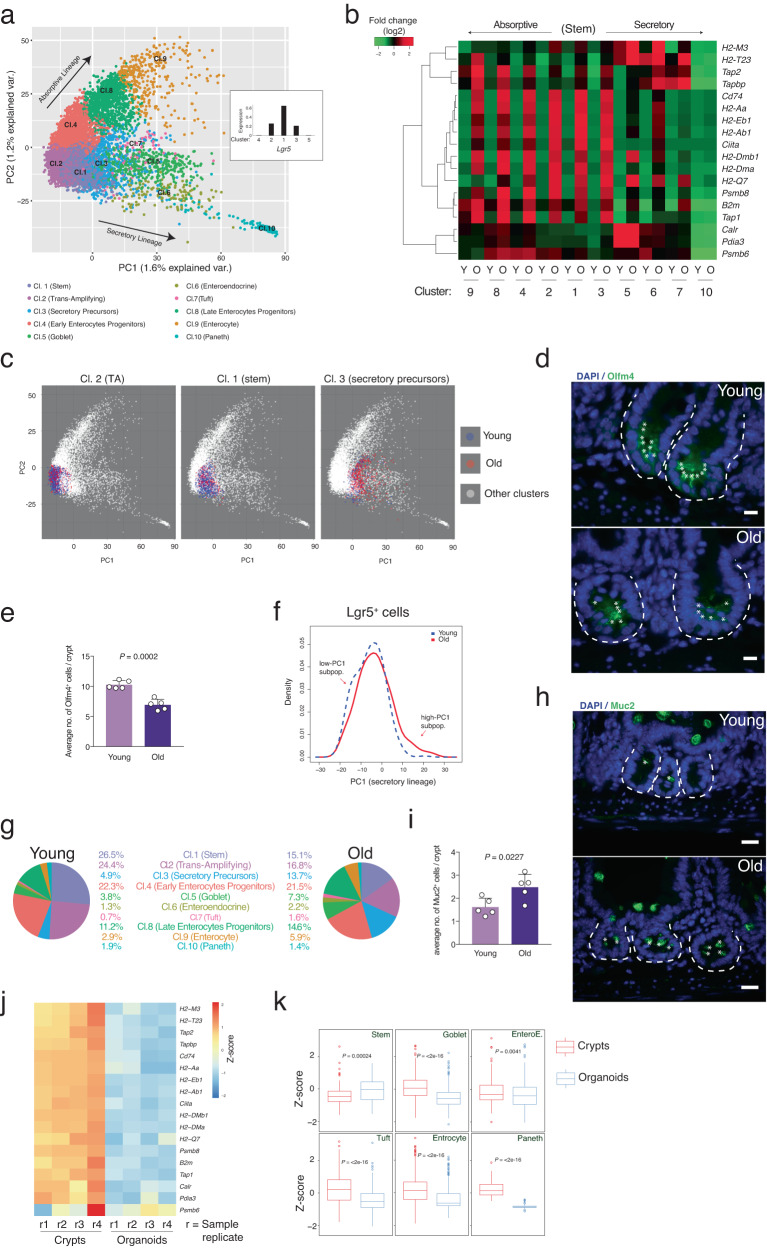
Intestinal crypt cell composition changes during aging. **a** Scatter plot of PC1 and PC2 values of the single cell RNA-seq (scRNA-seq) dataset (both the young and the old RNA-seq merged). Each cell is colored accordingly to its respective cluster (Cl.). Clusters are calculated by K-means. The small central panel represents the average of normalized read counts of the Lgr5 expression value in clusters 1–5. **b** Hierarchical clustering and heatmap of the expression level of the DEGs in the Antigen Presentation Pathway in the different clusters found in the scRNA-seq of young (Y) or old (O) mouse intestinal crypts. **c** Scatter plot of PC1 and PC2 values of the single cell RNA-seq (scRNA-seq) dataset (both the young and the old RNA-seq merged). Cells belonging to the indicated clusters are colored in blue (young-derived cells) or in red (old derived cells). **d** Representative pictures of anti-Olfm4 staining in young and aged proximal small intestine. Scale bar 20 µm. The dotted lines indicate the crypt structure. **e** The bar chart shows the average number of the Olfm4^+^ cells per crypts in the indicated groups. *n* = 5 mice per group were analyzed. Error bars represent the SD. *P* value was calculated by two-sided Welch’s *t* test. **f** Line-plot of the cell population density of the Lgr5-expressing cells (Lgr5^+^) along the PC1 axis measured by the scRNA-seq experiment. Old Lgr5^+^ cells show a shift in the PC1 profile with respect to the young ones. **g** Representative pie charts showing the percentage of cells belonging to each cluster in indicated ages. **h** Representative pictures of anti-Muc2 staining in the proximal small intestine of indicated ages. Scale bar 20 µm. The dotted lines indicate the crypt structure. **i** The bar chart shows the average number of the Muc2^+^ cells in the indicated ages. *n* = 5 mice per group were analyzed. Error bars represent the SD. *P* value was calculated by two-sided Welch’s *t* test. **j** Heatmap of the expression level (scaled by gene in the row) of the DEGs in the Antigen Presentation Pathway in 2 weeks cultured intestinal organoids. *n* = 4 mice per group. **k** Box plot of geneset enrichment analysis of the indicated gene datasets from each cluster as in **j** from in vivo proximal intestinal crypts and in vitro grown organoids. *P* value was calculated by two-sided Wilcoxon non-paired test.

The above data suggested that intestinal crypt and stem cells undergo massive changes in their transcriptomic signature during aging that could lead to impairment of the differentiation capacity as well as of the tissue homeostasis and functionality. To test whether aging would change the ISC identity and eventually alter the cell composition within crypts, we performed scRNA-seq of young and old freshly isolated crypt cells (Figs. 3a and S3a). We pooled intestinal crypts from the proximal part of the small intestine from 3 young mice (young sample) and 3 old mice (old sample). K-means clustering found ten main clusters in intestinal crypt cells defining the main well-known intestinal cell types (Fig. S3a, Supplementary Data S3) and principal components analysis (PCA) graphical visualization of the single cells nicely separated the 10 clusters on the basis of their stemness or differentiation lineage (Figs. 3a and S3b). Principal Component 1 and 2 (PC1 and PC2) distribution greatly reflected the differentiation status of the intestinal crypt cells, with PC1 representing the secretory lineage differentiation and PC2 the absorptive one (Figs. 3a and S3c, d). In agreement with the results from the bulk RNA-seq in crypts, scRNA-seq confirmed the overexpression of APP genes in almost all the intestinal cell types during aging, except the fully differentiated Paneth cells (Figs. 3b [cluster 10] and S3e, f). In accordance with the bulk RNA-seq of Lgr5^hi^ cells, scRNA-seq revealed an increase of Lgr5^+^ secretory precursor cells (cluster 3) in old intestinal crypts, while the other two Lgr5-expressing clusters (stem and TA cells) showed a reduction in cell number (Figs. 3c and S3g) indicating intestinal stem cell exhaustion in aged mice. Additionally, we observed, by using immunofluorescence staining, a decrease of Olfm4^+^ ISCs in intestinal crypts from old mice compared to young mice (Fig. 3d, e) and, by using FACS analysis, an analogous reduction of Lgr5^+^ ISCs (Fig. S3h). In accordance with the above-mentioned increase of transcriptionally primed secretory precursor population within Lgr5^+^ intestinal stem cells, the Lgr5-expressing cells from old mice were more broadly distributed along the PC1 axis, showing loss of a low-PC1 (PC1 parameter indicator of a transcriptome proper of cells differentiated towards the secretory lineage) subpopulation and the appearance of a high-PC1 subpopulation during aging (Fig. 3f). However, no difference can be seen along the PC2 axis for these Lgr5-expressing cells (Fig. S3i). In line with our results on increased secretory priming of the ISCs and also in line with previous studies^4,7^, there was an increase of secretory cells mainly Goblet, Enteroendocrine and Tuft cells within intestinal crypts during aging (Fig. 3g). To further validate this finding, we performed immunofluorescence staining for markers of secretory cells including Muc2 (for Goblet cells) and Chga (for Enteroendocrine cells) (Figs. 3h and S4a). The number of Goblet and Enteroendocrine cells within the intestinal crypts of old mice compared to young mice was almost doubled (Figs. 3i and S4b). Together, these data suggested that aging intestinal crypts exhibit a very strong increase of antigen presentation, a depletion of näive ISCs, and an increase in ISCs that are primed towards secretory lineage accompanied by increases in differentiated secretory cells.

Recent studies have shown that intestinal epithelium cells respond to signals coming from their milieu^13^. Intestinal epithelium cells, on one side, are in close contact with microbiome, and on the other side have crosstalk with lamina propria immune cells^37^. To test whether these two milieu components are responsible for the above-mentioned changes in aging intestinal crypt cells, we cultured freshly isolated intestinal crypts from old mice as organoids for 2 weeks. Surprisingly, our RNA-seq analysis of these organoids revealed a complete loss of expression of APP genes (Fig. 3j). Moreover, these organoids showed an increase in the expression of stem cell markers and a reduction in the expression of the secretory cell markers during two weeks of culture (Fig. 3k), revealing that the main phenotypes observed in aging crypts in vivo can be fully reverted when cultured in vitro. This result suggested that cell-extrinsic factors play a major role in driving aging-associated phenotypes in the intestinal epithelium. In line with the above-mentioned results, organoids from old mice showed a reduction in the expression of marker genes of multilineage-primed subpopulation (P2) and secretory precursors when compared to crypts in vivo (Fig. S4c). Together, these data suggested that intestinal crypts of aged mice respond to the surrounding aged niche by changing their cellular composition leading to a decrease in stem cells, an increase in secretory lineage cells, as well as an increase in the expression of APP genes.

### Lamina propria immune cell composition changes during aging

**Fig. 4:**
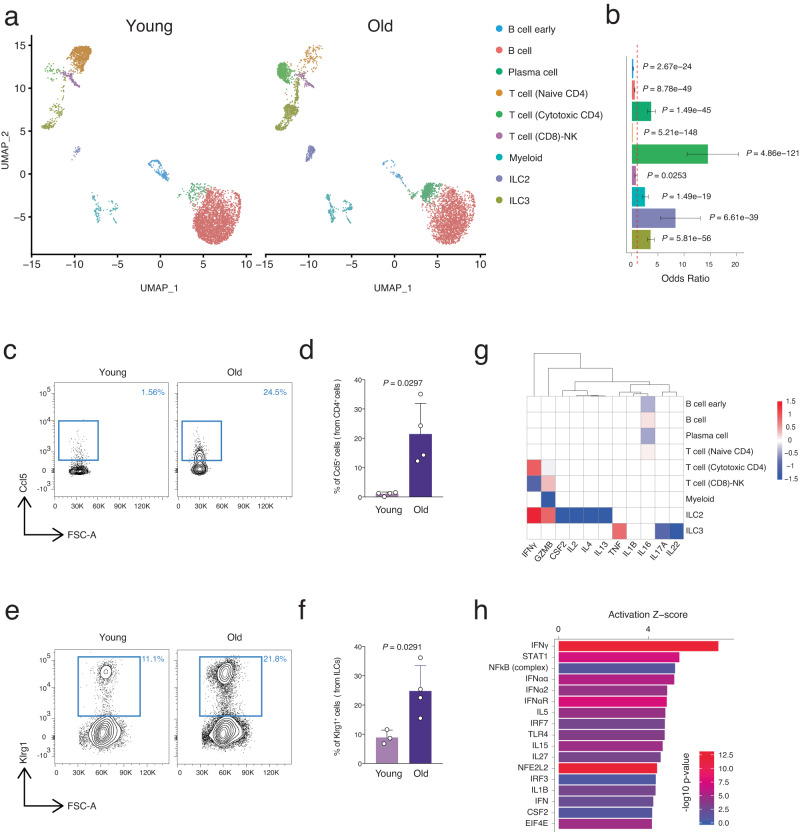
Aging lamina propria immune cell composition changes towards pro-inflammatory condition. **a** UMAP projection of all 8997 immune cells (4423 young cells and 4574 old cells) presenting nine different clusters, identified via shared nearest neighbor modularity optimization-based clustering algorithm, followed by merging of similar clusters. *n* $\ge$ 3 mice per group were analyzed. **b** Bar chart of odds ratio with 95% confidence interval showing the relative abundance of different immune cells changing in aging. *P* value was calculated by two-sided hypergeometric test. **c** Representative FACS plots showing percentages of CCL5^+^ CD4 T (cytotoxic) cells in the indicated ages. **d** The bar chart shows the percentage of CCL5^+^ cells from CD4 T cells in the indicated ages. *n* = 4 mice per group were analyzed. Error bars represent the SD. *P* value was calculated by two-sided Welch’s *t* test. **e** Representative FACS plots showing percentages of Klrg1^+^ ILC2s in the indicated ages. **f** The bar chart shows the percentage of Klrg1^+^ ILC2s in the indicated ages. *n* = 3 (young) and *n* = 4 (old) mice per group were analyzed. Error bars represent the SD. *P* value was calculated by two-sided Welch’s *t* test. **g** Hierarchical clustering and heatmap of gene expression for identified cytokines in our scRNA-seq datasets. **h** Upstream regulators associated with the DEGs from RNA-seq of intestinal crypts cells during aging. *X* axis shows the activation *Z* score of each upstream regulator, and color code shows enrichment *P* value (−log10). *P* value is calculated using the right-tailed Fisher Exact Test.

The above data suggest that aging of intestinal crypt cells may be driven by their niche factors since the main aging phenotypes were rescued during in vitro culture. Recently it has been shown that cytokines derived from *lamina propria* immune cells can alter the intestinal stem cell fate during infection^13,38,39^. To test whether aging would alter the *lamina propria* immune cell composition and whether their respective cytokines could attribute to the observed aging phenotype of intestinal crypt cells, we generated scRNA-seq data from freshly isolated, highly purified CD45^+^ FACS-sorted *lamina propria* immune cells from the proximal small intestine of young and old mice. To characterize *lamina propria* immune cell subsets in an unbiased manner and to find the change of these subsets during aging, we combined all cells from young and old mice, and by using their transcriptomic profiles we identified nine distinct and robust clusters (Fig. S5a). To associate each cluster with a known immune cell subset, we screened the most significantly upregulated genes of each cluster and compared them to previously reported immune cell subsets and to canonical markers of immune cells (Fig. S5b)^13,21,40^. Our clustering described four main categories of cells, the B cell family, the T cell family, Myeloid, and ILCs representing, respectively ~75%, ~15%, ~5%, and ~5% of all the *lamina propria* CD45^+^ cells from young mice (Fig. S5c). Within each family we observed significant cell-type shifts that occurred during aging (Figs. 4a, b and S5c). In the B cell compartment, as previously reported in bone marrow^41^, we were also able to detect an increase of plasma cells and a depletion of B and early B cells with age in the *lamina propria* of the small intestine (Figs. 4b, S5c). In the T cell compartment the number of naïve T cells dropped significantly and T cell composition shifted towards an increase in cytotoxic CD4 cells as previously reported in splenic tissue during aging (Fig. 4b, S5c)^21^. In contrast, the total myeloid cell population showed an increase with age. Lastly, the type 2 innate lymphoid cells (ILC2) showed the second highest enrichment immune cell population during aging in the intestinal *lamina propria* of mice after cytotoxic CD4 T cells, followed by ILC3 cells (Fig. 4b). To verify these findings, we focused our attention towards the two most enriched populations during aging, namely cytotoxic CD4 T cells and ILC2s, and to distinguish these two subsets of immune cells we utilized FACS analysis with Ccl5 and Klrg1 markers for cytotoxic CD4 T cells and ILC2s respectively (Fig. S5b, d). In agreement with the above results from scRNA-seq, FACS analysis revealed an increase of these two cell populations in the *lamina propria* of old mice compared with young mice (Fig. 4c–f). Together, these data indicated that intestinal *lamina propria* immune cells undergo cell-type composition alterations during aging, experiencing a significant increase of the pro-inflammatory cytotoxic CD4 T cells and the ILC2 compartment.

Next, the aging-related mRNA expression changes of cytokines were determined in the different subtypes of immune cells from the intestinal *lamina propria* (Fig. 4g). Among the well-known cytokines produced by different immune cells, IFNγ expression level was increased during aging, specifically in the ILC2 and cytotoxic CD4 T cells, whose number also showed a significant increase with aging (Fig. 4b). To verify the link between the cytokines produced by cytotoxic CD4 T cells and the ILC2s and the alterations observed in the intestinal epithelium during aging, we executed a predictive analysis of the upstream regulators of the DEGs observed in intestinal crypt cells during aging (analysis shown in Fig. 1). Interestingly, IFNγ was predicted to be the top upstream regulator driving the transcriptional changes induced by aging in intestinal crypt and stem cells (Fig. 4h). Importantly, previous studies have shown that IFNγ can regulate intestinal crypt cell homeostasis and the MHCII gene expression on epithelial cells^42–45^. These results suggested that the aging intestinal niche leads to an accumulation of pro-inflammatory immune cells including cytotoxic CD4 T cells and ILC2s in the intestinal *lamina propria*, which secrete elevated levels of cytokine prominently involving IFNγ that may impinge on intestinal epithelium homeostasis and function.

### IFNγ treated organoids mimic in vivo intestinal crypt cells aging

**Fig. 5:**
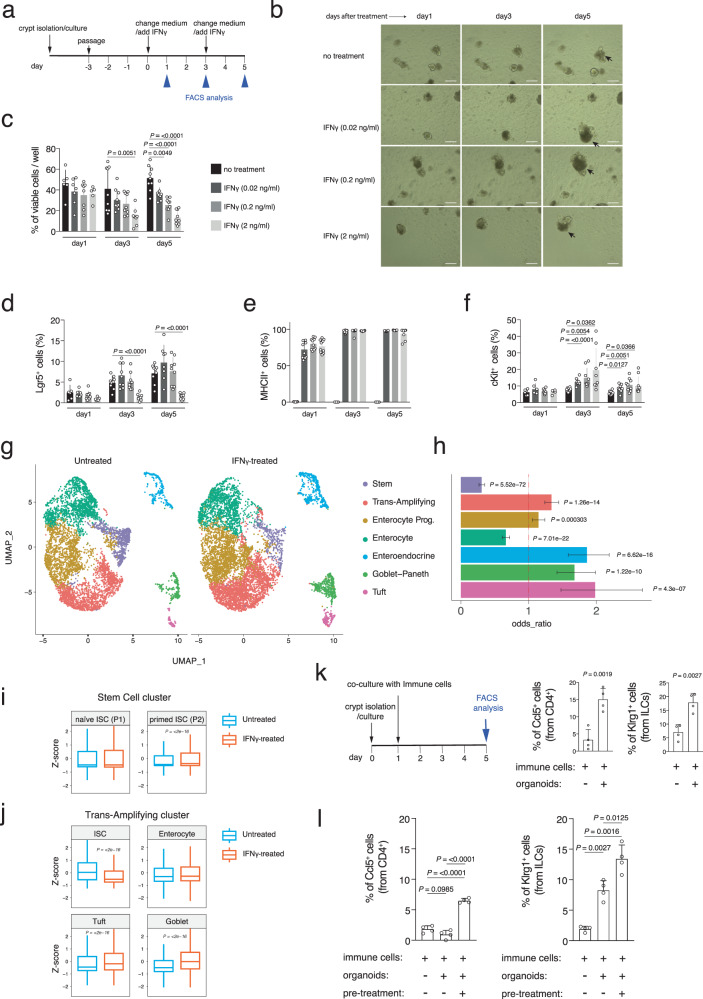
IFNγ treatment of intestinal organoids mimics the intestinal crypt aging phenotype. **a** Experimental scheme of IFNγ treatment of intestinal organoids. **b** Brightfield images of intestinal organoids that were treated with different concentration of IFNγ for 5 days. Non-treated cultures served as control. Apoptotic cells are indicated by arrow. Scale bar 200 µm. **c**–**f** Time-course analysis of organoids stimulated with different concentration of IFNγ for 5 days. Dissociated organoid cells were analyzed by FACS after cKit, MHCII, and DAPI staining. **c** viable cell percentage, **d** Lgr5^+^ cell percentage, **e** MHCII^+^ cell percentage, and **f** cKit^+^ cell percentage. Each dot represents one mouse. *n* = 9 mice per group were analyzed. Error bars represent the SD. *P* value was calculated by two-sided Welch’s *t* test. **g** UMAP projection of organoid culture derived EpCAM^+^ cells presenting seven different clusters, clustered via shared nearest neighbor modularity optimization-based clustering algorithm. Clusters were identified to epithelial cell types by using expressed markers. *n* $\ge$ 3 mice per group were analyzed. **h** Bar chart of odds ratio with 95% confidence interval showing the relative abundance of different epithelial cells changing after IFNγ treatment. *P* value was calculated by two-sided hypergeometric test. **i**, **j** Boxplots of geneset enrichment analyses of the indicated gene datasets in the in vitro treated intestinal organoids treated with 2 ng/ml IFNγ for 24 h. *P* value was calculated by two-sided Wilcoxon non-paired test. **k** The bar charts show the percentage of Ccl5^+^ CD4 Cytotoxic T cells (left panel) and Klrg1^+^ ILC2s (right panel) in the indicated conditions. Organoids were derived from old mice. *n* = 4 mice per group were analyzed. Error bars represent the SD. *P* value was calculated by two-sided Welch’s *t* test. **l** The bar charts show the percentage of Ccl5^+^ CD4 Cytotoxic T cells (left panel) and Klrg1^+^ ILC2s (right panel) in the indicated conditions. Organoids were derived from young mice. *n* = 4 mice per group were analyzed. Error bars represent the SD. *P* value was calculated by two-sided Welch’s *t* test.

The above data indicated that during aging and due to changes in the *lamina propria* immune cells, intestinal crypts encounter a pro-inflammatory environment that eventually leads to the expression of APP genes and changes in the crypt cell composition (stem cell decrease / secretory cells increase). Moreover, IFNγ produced by aged *lamina propria* immune cells was identified as the upstream regulator of these changes in intestinal crypt cells during aging. Since these aging-induced changes in intestinal crypts were lost in vitro (Fig. 3j, k), we decided to employ in vitro organoid culture for functional experiments (Fig. 5a–f). We applied different concentrations of IFNγ (0.02, 0.2, and 2 ng/ml) at day 3 of culture after passaging the organoids and monitored them in the following days (Fig. 5a, b). FACS analysis was performed to quantify the cell viability (DAPI^+^ cells), the intestinal stem cells (Lgr5^+^), APP gene expression (MHCII^+^), and the secretory precursors/cells (cKit^+^). cKit expression was employed as a marker of secretory precursors as it becomes upregulated in stem and TA cells primed to differentiate and is strongly expressed in all the secretory lineage cells (Fig. S6a, b)^13,34^. As previously shown^44,46^, exposure of organoids to IFNγ led to an increase in apoptotic cells and a reduction in cell viability in a time- and dose-dependent manner (Fig. 5b, c). Applying 2 ng/ml of IFNγ significantly increased the dead cell percentage after 3 days in culture and as expected, reducing the IFNγ concentration to 0.02 ng/ml led to a better survival of the organoids. Interestingly, low dose IFNγ exposure led to an increase in organoid cell number possibly due to the increase of intestinal organoid cell proliferation in culture (Figs. 5b and S6c). Accordingly, in presence of low doses of IFNγ, the Lgr5^+^ intestinal stem cells could proliferate and expand similar to untreated samples, while high dose of IFNγ led to a strong depletion of Lgr5^+^ intestinal stem cells in vitro after 3 days of exposure (Fig. 5d). In agreement with the Lgr5^+^ cells, the number of the Olfm4^+^ cells (another marker for intestinal stem cells) showed strong reduction of stem cell population after IFNγ treatment (Fig. S6d). Interestingly, exposure of organoids to IFNγ, even with a low 0.02 ng/ml dose, could strongly induce APP gene expression as evident by an increase in MHCII^+^ cell percentage to 70% after one day and to 100% at day 3 (Fig. 5e) as previously reported^44^. Moreover, IFNγ significantly increased secretory (cKit^+^) cells in culture after 3 and 5 days with all the tested concentrations (Fig. 5f). Together, these data suggest that IFNγ could mimic intestinal aging phenotypes in organoid culture. We also tested long-term (3 weeks) treatment of organoids with low doses IFNγ and we observed that, eventually, low doses of IFNγ can drive the intestinal aging phenotypes observed in vivo (Fig. S6e). To validate our findings and to understand early transcriptional changes after IFNγ exposure, we conducted scRNA-seq from old mice-derived organoids treated with IFNγ for 24 h. Clustering of the organoid cells using the UMAP method could distinguish 7 clusters of cells as previously described^13^ (Fig. 5g). In agreement with the aforementioned results, scRNA-seq showed an increase in secretory cells after IFNγ exposure (Figs. 5h and S6f). Moreover, we observed stem cell depletion and a slight increase in transit-amplifying (TA) cells suggesting that ISCs may be stimulated by IFNγ to leave stemness and enter into the cell cycle with a secretory-lineage fate (Figs. 5h and S6f).

By using the above-mentioned P1 and P2-specific gene datasets, we were able to dissect early transcriptional responses in the stem and TA cell clusters (Fig. 5i, j). Stem cells from in vitro grown organoids, after IFNγ treatment, showed a significant increase in the multilineage-primed subpopulation (P2) when compared to the naïve stem cell population (P1) (Fig. 5i). TA cells, probably containing many IFNγ-activated ISCs that left the stem cell cluster, showed strong reduction of ISC-related genes and enrichment of transcripts of differentiated secretory cells, like Tuft or Goblet cells (Fig. 5j).

Similar to our scRNA-seq data from intestinal crypt cells during aging (Fig. 3), scRNA-seq of IFNγ treated organoids revealed an increase of MHCII gene expression in almost all the cell types of intestinal organoid (Fig. S6g). Interestingly, when plotting all the genes of the APP (Fig. S6h), we observed that different cell types overexpressed different subsets of the genes in response to IFNγ treatment; this suggested that each cell type may have different immunological functions upon stimulation by immune cells. Altogether, these experiments indicated that IFNγ triggers the ISC activation and exit from a stem state towards cells that are more proliferating and transcriptionally primed towards the secretory lineage, expressing MHCII genes. It has been recently observed that naïve immune cells can be activated by MHCII^+^ ISCs^13^. To understand whether MHCII+ epithelial cells could expand the CD4 T and the ILC2 immune cells, we co-cultured immune cells from the *lamina propria* with intestinal organoids freshly isolated from old mice and observed an expansion of both the two immune cell populations (Fig. 5k). Moreover, immune cells expanded in vitro also when cultured with intestinal organoids from young mice but pre-treated with IFNγ to overexpress MHCII genes, confirming the role of IFNγ in mediating epithelial-immune cell communications (Fig. 5l). Our data indicate that while acute exposure to IFNγ leads to a severe stem cell apoptosis and depletion, chronically low exposure to IFNγ may drive the phenotypes described herein aging intestinal crypts, in particular the stem cell reduction, the secretory lineage skew and, finally, the conversion to an antigen-presenting tissue that triggers an immune response.

### Blocking IFNγ restores aging-induced alterations in the intestinal tissue

**Fig. 6:**
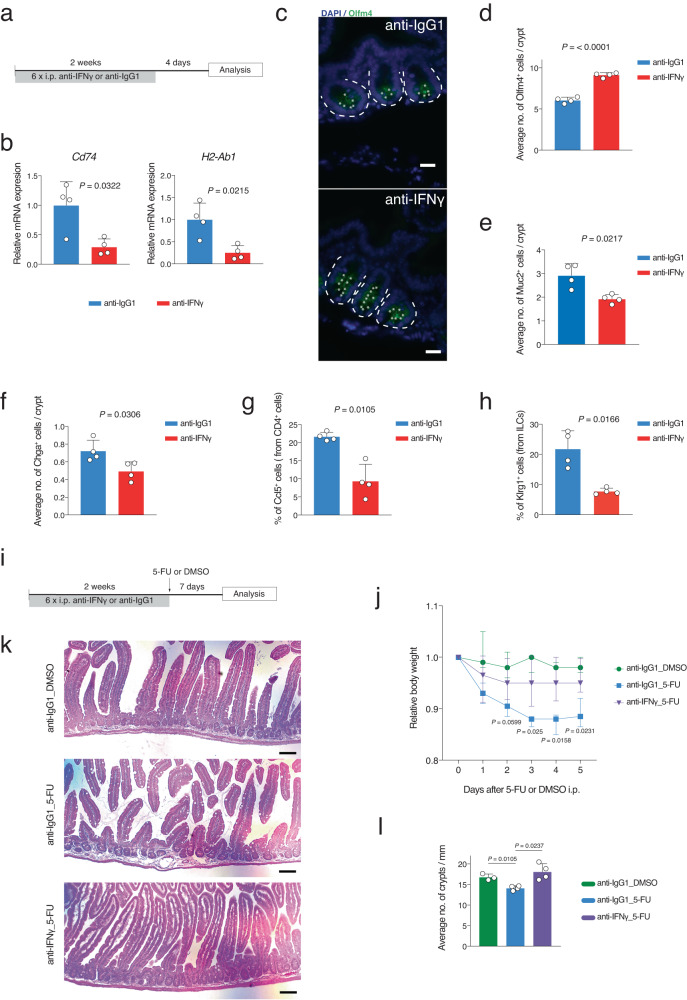
In vivo IFNγ inhibition in aged mice rescues the aging-induced alteration in intestinal crypt cells. **a** Schematic representation of IFNγ inhibition experiment. Anti-IFNγ antibody (25 mg/kg) was injected intraperitoneally (i.p.) for two weeks (3 injections per week) in old mice. Mice were sacrificed for organ harvest 4 days after the last injection. Anti-IgG1 antibody were used as control. **b** qRT-PCR validation of *Cd74* and *H2-Ab1* gene expression levels in indicated conditions. *n* = 4 mice per group were analyzed. Error bars represent the SD. *P* value was calculated by two-sided Welch’s *t* test. **c** Representative pictures of anti-Olfm4 staining in indicated conditions. Scale bar, 20 µm. The dotted lines indicate the crypt structure. **d**–**f** The bar charts show the immunofluorescence quantification of the Olfm4^+^, Muc2^+^, and Chga^+^ cells in the indicated conditions. *n* = 4 mice per group were analyzed. Error bars represent the SD. *P* value was calculated by two-sided Welch’s *t* test. **g**, **h** The bar charts show the percentage of Ccl5^+^ CD4 T (cytotoxic) and Klrg1^+^ ILC2 cells quantified by FACS analysis in the indicated conditions. *n* = 4 mice per group were analyzed. Error bars represent the SD. *P* value was calculated by two-sided Welch’s *t* test. **i** Schematic representation of the in vivo IFNγ inhibition experiment for regeneration analysis. Anti-IFNγ antibody (25 mg/kg) was injected intraperitoneally (i.p.) every second day. 3 injections per week for 2 weeks were performed. One day after the last injection, 5-fluorouracil (5FU; 150 mg/kg) or DMSO as control were intraperitoneally (i.p.) injected. **j** Body weights of treated old animals shown as mean ± SD. *n* = 4 mice per group were analyzed. *P* value was calculated by two-sided Welch’s *t* test. *k* Representative picture of H&E-stained longitudinal sections of the proximal part of the intestine from indicated conditions. Scale bars 100 µm. **l** The bar chart shows the average number of crypts per millimeter of the proximal part of the small intestine of the indicated groups. *n* = 4 mice per group were analyzed. Error bars represent the SD. *P* value was calculated by two-sided Welch’s *t* test.

Taken together, the data shown above suggest that an aging-associated alteration in the intestinal niche towards a pro-inflammatory environment contributes to the impairment of the ISC identity and subsequent tissue maintenance and functionality through IFNγ action. To test this hypothesis and to ameliorate the observed aging-associated phenotypes including the impaired regeneration capacity of aged intestine^4,6,11^, we decided to inhibit IFNγ in vivo in 24-month-old mice using anti-mouse IFNγ antibody or anti-IgG1 antibody as a control (Fig. 6a). Blocking of IFNγ for two weeks led to a significant reduction in mRNA expression of APP genes from intestinal crypt cells (Fig. 6b). To analyze whether this treatment had some effect on the stem cell number of aged intestinal crypt cells, we performed Olfm4 immunofluorescence staining that revealed an increase in number of Olfm4^+^ stem cells after IFNγ inhibition (Fig. 6c, d). Immunofluorescence staining also revealed that the number of Goblet cells (Muc2^+^) and Enteroendocrine cells (Chga^+^) were also restored to the level of the young (e.g., 1.8 Muc2^+^ cells per crypt in young in Fig. 3i) (Fig. 6e, f). RT-qPCR quantification of the expression levels of marker genes of ISCs and secretory cells, confirmed the above findings (Fig. S7a). Interestingly, FACS analysis of the *lamina propria- * associated immune cells showed a significant reduction in cytotoxic CD4 T cells and ILC2s after blocking IFNγ (Fig. 6g, h)^13^. To substantiate the evidence that two weeks IFNγ inhibition impacts on intestinal homeostasis, the regeneration capacity of the intestine was analyzed after 5-fluorouracil (5-FU) induced injury of the intestinal epithelium^6,47,48^.

2-years old mice were treated with anti-IFNγ or anti-IgG1 antibodies for 2 weeks prior to the regeneration challenge with 5-FU or dimethyl sulfoxide (DMSO) as control (Fig. 6i). Of note, pretreatment of aged mice with anti-IFNγ antibody ameliorated the body weight loss in response to 5-FU treatment, especially at later time points (Fig. 6j). To analyze the impact of IFNγ blockage on 5-FU induce alterations in intestinal epithelium at histological level, the number of intestinal crypts was determined in the treatment groups. While 5-FU treatment led to a significant reduction in intestinal crypt numbers, IFNγ blockage completely prevented this crypt atrophy (Fig. 6k, l). Together, these results support a functional role of IFNγ in increasing the APP gene expression, aging-related changes in crypt cell composition, and in impairment of the regeneration capacity of the aged intestinal tissue.

### Stat1 activation downstream of IFNγ mediates ISCs aging phenotype

**Fig. 7:**
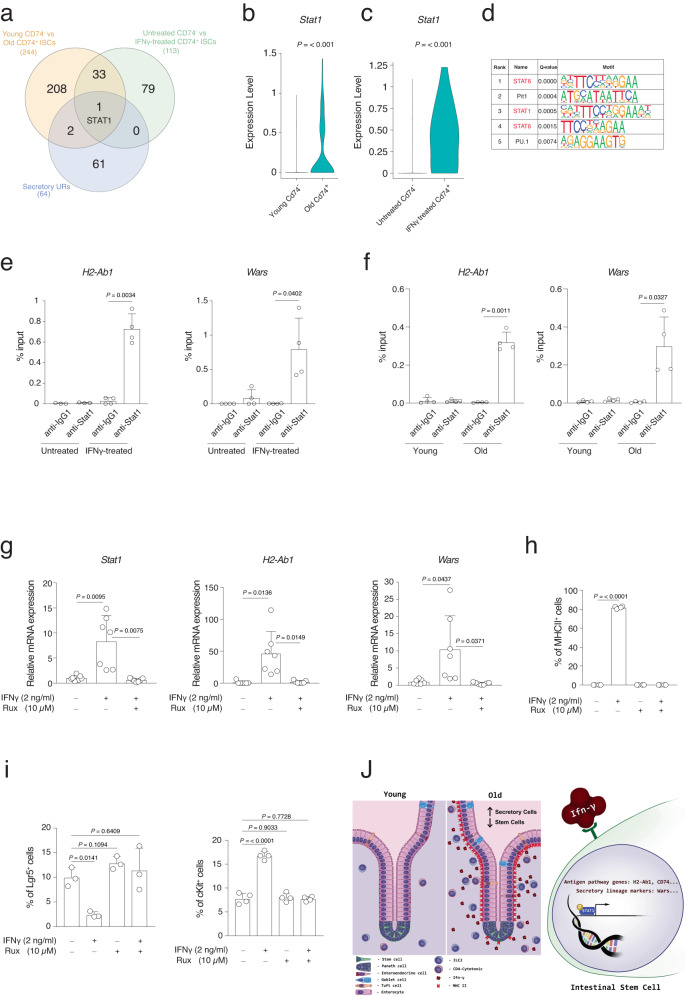
Stat1 transcription factor mediates the IFNγ effect during aging. **a** Gene set A: DEGs using young CD74^−^ intestinal stem cell versus old CD74^+^ intestinal stem cell in crypts, Gene set B: DEGs using control CD74^−^ intestinal stem cell versus IFNγ treated CD74^+^ intestinal stem cell in organoids, Gene set C: IPA-predicted upstream regulators for secretory lineage. **b**, **c** Box plot of Stat1 expression in the indicated groups. *P* value was calculated by two-tail Wilcoxon rank sum test. **d** Transcription factor and binding motif prediction on the promoters of the gene markers of the secretory cells in intestine. Top5 significant predicted motifs (*q* value < 0.001) are shown, indicating STAT6 and STAT1 were significantly enriched to bind secretory lineage gene promoters. *P* values was calculated by two-tail binomial distribution. *Q* value was Benjamini-adjusted *p* value. **e**, **f** ChIP-qRT-PCR analysis of the ChIP of Stat1 on *H2-Ab1* and *Wars* promoters in **e** organoids treated or untreated with IFNγ (2 ng/ml) for 24 h or **f** intestinal crypts of young or old mice. Each dot represents one mouse. *n* = 4 mice per group were analyzed. Error bars represent the SD. *P* value was calculated by paired two-tail *t* test. **g** qRT-PCR analysis of *Stat1*, *H2-Ab1,* and *Wars* gene expression level in the indicated conditions. *n* = 7 mice per group were analyzed. Error bars represent the SD. *P* value was calculated by two-sided Welch’s *t* test. **h** The bar chart shows the percentage of MHCII^+^ cells in the indicated conditions. Organoids treated with IFNγ (2 ng/ml) with or without Ruxolitinib (Rux) for 3 days. *n* = 4 mice per group were analyzed. Error bars represent the SD. *P* value was calculated by two-sided Welch’s *t* test. **i** The bar chart shows the percentage of Lgr5^+^ cells and cKit^+^ cells in the indicated conditions. *n* = 4 mice per group were analyzed. Error bars represent the SD. *P* value was calculated by two-sided Welch’s *t* test. **j** Graphical abstract modeling aging-induced alteration in intestinal tissue.

The above-described data indicated that ISC functional decline during aging is due to the activation of downstream targets of IFNγ, which is produced by immune cells located in the aged intestinal lamina propria. Therefore, we aimed to find the transcription factors that mediate the IFNγ-induced aging phenotype in the ISCs with a particular focus on the secretory lineage skew. Using Cd74 as a marker of IFNγ stimulation, single-cell RNA-seq was employed to analyze freshly isolated intestinal epithelial cells from unperturbed intestine. Using this approach, it was possible to separate epithelial cells that were activated by IFNγ (Cd74^+^ cells) or not (CD74^−^) at the time of cell extraction. We overlapped DEGs from Cd74^−^ ISCs of young mice *versus* Cd74^+^ ISCs of old mice with DEGs from Cd74^-^ ISCs of untreated organoids *versus* Cd74^+^ ISCs of IFNγ-treated. Further crossing of these two datasets with IPA-predicted upstream regulators for secretory lineage marker genes (Fig. 7a) resulted in the only transcription factor Stat1 being present in all three datasets. Remarkably, its expression level was found to increase during aging, as well as after IFNγ treatment of intestinal organoids (Fig. 7b, c).

To further verify the role of Stat1 in mediating the downstream effects of IFNγ and altering the ISC transcriptional identity, we predicted transcription factors having binding motifs on the promoters of marker genes of secretory lineage that were identified in our scRNA-seq analysis and elsewhere^34,35^. Based on our prediction, Stat transcription factor family including Stat1 and Stat6 were among the top5 transcription factors binding to the promoter of the secretory genes that were found upregulated in aging (Fig. 7d). In fact, Stat1 was the second upstream regulator associated with the DEGs from the RNA-seq of intestinal crypts cells during aging (Fig. 4h). We investigated the binding ability of the Stat1 transcription factor on promoters of IFNγ target genes after IFNγ treatment on intestinal organoids. Interestingly, chromatin immunoprecipitation (ChIP) assay revealed that Stat1 binds to the promoter of the APP genes like *H2-Ab1* and the secretory marker genes like *Wars* (Fig. 7e). Interestingly, Stat1 was found to bind these promoters also in intestinal crypts of old mice (Fig. 7f). To mechanistically prove the functional role of Stat1 as a downstream transcription factor of the IFNγ-induced effect in intestinal crypt cells we utilized the inhibitor of Stat1 signaling, Ruxolitinib, which prevents phosphorylation of Stat1 by IFNγ in intestinal crypt cells^45^. In accordance with previous studies^49^, Ruxolitinib can inhibit the Stat1 mRNA expression in organoids after IFNγ treatment and at the same time, remarkably, could block the expression of APP genes like *H2-Ab1* and the secretory marker genes like *Wars* (Fig. 7g). In line with these data, FACS analysis for quantifying MHCII^+^ cells also showed that Ruxolitinib can prevent the expression of APP genes in organoids after IFNγ exposure (Fig. 7h). Furthermore, FACS analysis also showed that inhibiting Stat1 signaling rescues the decrease in Lgr5^+^ ISCs number and the increase of the secretory precursors/cells (cKit^+^ cells) in organoid culture after IFNγ treatment (Fig. 7i). Of note, organoids treated with IFNγ and Ruxolitinib did not show any adverse effect on the organoid’s growth, apoptosis, proliferation, and differentiation (Fig. S7b–e). To further validate the role of Stat signaling on intestinal organoids, we used another Jak/Stat inhibitor (Baricitinib) and we observed a significant rescue, following IFNγ treatment, of (1) the MHCII genes overexpression, (2) stem cell markers reduction, and (3) secretory cell markers increase (Fig. S7f). Altogether, these results indicate the importance of Stat1 signaling downstream of IFNγ in mediating ISC fate alteration during aging (Fig. 7J).

## Discussion

Aging is a complex multifactorial process that negatively affects the functionality of various tissues, including the gut system. Intestine from old individuals often show a loss of functionality and different aging-associated diseases, including cancer. The current study provides experimental evidence that aging-induced IFNγ production by the lamina propria immune cells leads to transcriptional and cell composition alterations in intestinal crypts as well as ultimately to impairment of the intestine regenerative capacity.

Our study showed an increase of the transcriptional variance in intestinal crypt cells from aged mice, a finding that has previously been observed in other tissues like pancreas, liver, and heart, suggesting that increasing variability in gene expression might be a hallmark of aging^24,50,51^. As previously reported in invertebrates, like *D. melanogaster*^52,53^, our study also shows gender-specific gene regulation alterations during intestinal aging. In line with this finding, while 26-month-old male mice showed a slight amelioration of the aging-associated transcriptional changes with respect to 18-month-old male mice, female mice showed a continuous trend of age-related transcriptional regulation. This male-specific U-shape pattern, whose cause still remains not completely understood, has been already detected in other tissues and animals^23,24^, but it has been never observed in a gender-specific fashion. Additionally, we observed specific metabolic and signaling pathways that were strongly enriched in a gender-dependent manner in old mice. For example, mTOR and Sirtuin signaling pathways, two well-known pathways in the aging field^54,55^, were differentially enriched and regulated between old female and male intestinal crypts. In contrast to metabolic and signaling pathways, inflammation-related pathways showed a significantly higher gender correlation suggesting that the intestinal epithelial cells from both genders are responding to similar inflammatory cues during aging.

We also observed ISC-specific alteration of signaling and metabolic pathways, that have been demonstrated to regulate stem cell functionality in the intestine or in other aging compartments (e.g., Aryl Hydrocarbon, IGF-1, p53, calcium, and fatty-acid oxidation signaling)^5,31,32,56^. Remarkably, other metabolic and signaling pathways were more enriched at crypt level, but not detectable at ISC level. This evidence suggests that aging may differently affect the transcriptional landscape of each cell type in an organ. Nevertheless, the same inflammation-related pathways altered in all the crypt cells, were also present in the purified intestinal stem cells indicating that they may be altered at the stem cell level and then maintained in all the daughter cells.

Characterization of the gender-, age- and cell-type specific mechanisms highlights the need to take into consideration these parameters in basic research as well as intrinsic organismal factors (especially age and gender) in clinical praxis.

Studies on young mice reported that APP genes, especially the MHCII genes, play an important role in ISC fate decision and in remodeling crypt and *lamina propria* cell compositions upon infection and in disease models^13,45^. Our results showed that during aging there is a strong upregulation of the MHCII genes in all the intestinal crypt cells. This upregulation suggests that during aging the intestinal epithelium becomes a non-conventional antigen-presenting organ that could affect the surrounding immune cells, altering their signaling and their functionality and triggering a positive feedback loop^13,45^.

According to here presented results, old Lgr5^hi^ cells show an increase of markers of secretory precursors and of fully differentiated secretory cells providing transcriptional evidence for an increase of an Lgr5^hi^ secretory-primed stem cell population during aging. Single-cell analysis confirmed the aging-associated increase of the secretory precursor cell population. Our data also indicated the presence of a Lgr5^hi^ stem subpopulation of naive cells in young animals with a strongly silent secretory transcriptome, possibly to maintain a higher number of self-renewing stem cells and their correct differentiation programs. However, upon aging the stem cell compartment becomes more prone to promote secretory differentiation, which perhaps is to respond to the loss of appropriate niche or systemic signaling or the increase of infections due to the loss of the intestinal barrier function. Our study can explain previous observations of an increased number of Goblet, Tuft, or Paneth cells in the intestinal epithelium of aged organisms^4–10^.

We found that intestinal crypt cells in vivo are exposed to inflammatory cues from their niches, especially from *lamina* *propria* immune cells, that trigger transcriptional and differentiation-associated responses. Organoids derived from aged animals revert these transcriptional alterations and revert changes in their cellular composition indicating that the extrinsic factors are responsible for the aging phenotype of the intestinal epithelium. Previous studies did not reveal the here identified prominent role of immune cells derived IFNγ on intestinal crypt cells, especially on ISCs, during aging. Accumulation of cytotoxic CD4 T cells and type 2 innate lymphoid cells at the *lamina propria* during aging indicate a pro-inflammatory environment in the intestinal tissue possibly due to intestinal barrier dysfunction that occurs during aging^57^. Our data showed an increase in secretory cells, especially Goblet cells, which could lead to a strengthening in the intestinal barrier by increased production of mucin proteins. Additionally, the observed increase in Tuft cells could lead to the expansion/recruitment of type 2 innate lymphoid cells to the site of inflammation for repairing (protecting) the intestinal barrier^58,59^. Given that in vivo blocking of IFNγ reduces the expression of MHCII genes in intestinal crypt cells and at the same time lowers the number of cytotoxic CD4 T cells in *lamina propria* of aged mice, it is conceivable that, as previously shown in infections and disease models, expression of MHCII genes by epithelial cells during aging is important for expansion of this T cell population in intestinal *lamina propria*^13,27^. It is tempting to speculate that, during aging, the crosstalk between immune cells and epithelial cells through IFNγ-Stat1-MHCII axis is activated, and creates a feed-forward loop for expansion of immune cells and expression of MHCII by intestinal crypt cells. Remarkably, previous studies did not reveal the here identified, noticeable role of Stat1 transcription factor in transcribing the MHCII genes and at the same time controlling the secretory lineage priming in intestinal stem cells during aging.

Altogether, our data represents an unprecedented overview of the transcriptional and cellular composition landscape of intestinal tissue during aging. Importantly, we identified IFNγ as the key player in the intestinal alterations that affect tissue functions and regeneration in aging. We demonstrated that blocking IFNγ in vivo fully rescues aging-associated intestinal phenotypes and loss of regeneration, elevating IFNγ as a very promising therapeutic target for the treatment of complications of human aging-associated intestinal diseases, including intestinal cancer.

## Methods

### Mice

Young (2–4 months old) and old (18–26 months old) male and female wild-type C57BL6/J, Lgr5-ki-eGFP-creER, and Olfm4-ki-eGFP-creER mice were group housed and maintained in a Specific Opportunist Pathogen Free (SOPF) animal facility in Fritz Lipmann Institute with 12 h of light/dark cycle and fed with a standard mouse chow at Temperature 20 ± 2 °C, rlH 55% ± 15. Experiments were conducted according to protocols approved by the state government of Thuringia Thüringer Landesamt für Verbraucherschutz (TLV) authority (licenses number: TG/J-0002858/A; TG/J-0003616/A; TG/J-0003681/A; FLI-17-109; FLI-18-005, FLI-20-005).

### Small intestine crypt isolation

Small intestinal crypts were isolated using the established protocol^60^ with some modifications. Briefly, mouse small intestine was dissected, washed in cold PBS. The villi-free intestinal pieces (2 cm) were washed with cold PBS and transferred to 5 mM EDTA/PBS, followed by two 30-min incubations at 4 °C on a rotator. The tissue was transferred to fresh cold PBS and manually shaken for 30 sec. The crypt solution was filtered using a 70 µm cell strainer and centrifuged at 450 × *g* for 5 min at 4 °C. Isolated crypts were immediately used or snap-frozen in liquid nitrogen and stored at −80 °C for further experiments. For RNA isolation, the crypts were immediately resuspended in QIAzol Lysis Reagent (Qiagen) and stored at −80 °C.

### Intestinal stem cells isolation and sorting

To isolate the Lgr5-eGFP and Olfm4-eGFP ISCs, the freshly isolated crypts were dissociated with the mixture of 18 ml TrypLE Express, 2 ml of 10× DNase I Buffer (100 mM Tris-HCl pH 7.5, 25 mM MgCl_2_, 5 mM CaCl_2_) and 1 ml DNase I (10 mg/ml) for 30 min at 37 °C with brief vortexing every 10 min. The single-cell suspension was then passed through a 20 µm cell strainer and centrifuged at 800 × *g* for 5 min at 4 °C. Cell pellet was resuspended in 3 ml FACS staining medium (FSM) containing PBS supplemented with 2% Fetal Bovine Serum, 2.5 mM EDTA, 10 µM Y27632 and DAPI (1:1000). The single cell suspension was applied to FACS LSRII (BD Biosciences) and the Lgr5-eGFP^hi^ or Olfm4-eGFP ISCs were sorted for downstream analysis as previously described^61^.

### Organoid culture

Small intestinal organoids were cultured according to the established protocol^62^. Briefly, isolated crypts were mixed with Matrigel and plated in 24-well plate. After polymerization of Matrigel, crypt culture medium (Advanced DMEM/F12, 1× Glutamax, 10 mM HEPES, N2 supplement (1:100), B27 supplement (1:50), 0.5 U/mL penicillin/streptomycin, 50 ng/mL mouse recombinant epithelial growth factor, 100 ng/mL mouse recombinant Noggin, and 500 ng/mL human recombinant R-spondin1) was added. For IFNγ treatment, two weeks grown organoids were passaged and at day 3, IFNγ (2 ng/ml) was added to them. After 24 h the organoids were collected, washed with PBS and processed for single-cell RNA-seq. For blocking of Stat1 signaling, organoids were treated with IFNγ 2 ng/ml with or without Ruxolitinib (10 µM) for 3 days and then collected for FACS or qRT-PCR analysis. IFNγ was used at the concentration of 0.2 ng/ml for re-seeding experiment and for Baricitinib (2 µM) experiment.

### Annexin V staining

After single cell preparation from organoids as described above, cells were resuspended in 200 µl of 1× binding buffer and 1 μl of APC Annexin V from apoptosis detection Kit from BD Bioscience following incubation at RT for 15 min. After washing with 1× binding buffer, cells were resuspended in 1× binding buffer (100 µl) and analyzed using FACSAriaII (BD Biosciences) and data were analyzed using FlowJo software.

### BrdU proliferation analysis

BrdU (10 µM) was added to organoids 6 h before harvesting. After single cell preparation from organoids as described above, the BrdU flow kit from BD Bioscience was used to quantify the percentage of proliferating cells in organoid culture. Cells were analyzed using FACSAriaII and FlowJo software was used for analysis.

### Co-culture experiment

IFNγ treated (0.2 ng/ml for 2 days) or untreated organoids were co-cultured with freshly isolated and sorted Cd45^+^ cells from young mice for 3 days. Roughly 2 × 10^5^ Cd45^+^ cells were mixed with 100 organoids and resuspended in Matrigel (30% final concentration). Then, immune cells were isolated from culture, and after staining, they were analyzed using FACSAriaII. FlowJo software was used for the percentage calculation of different population of immune cells.

### In vivo blocking of IFNγ

Old mice were injected intraperitoneally with anti-mouse IFNγ (25 mg/kg) or anti-IgG1 antibody for two weeks (3 injections per week). Mice were sacrificed for organ harvest 4 days after the last injection. For regeneration experiment, after blocking of IFNγ, 5-fluorouracil (5-FU) (150 mg/kg) or DMSO as control were intraperitoneally (i.p.) injected one day after the last injection and mice were sacrificed for organ harvest 7 days later.

### RNA and DNA isolation

Total RNA from crypts was isolated using QIAzol Lysis reagent (Qiagen) followed by isopropanol precipitation. RNA from Lgr5-eGFP^hi^ ISCs sorted by FACS were isolated using ZR-Duet™ DNA/RNA MiniPrep Plus Kit (Zymo Research) following the manufacturer’s instructions. Isolated RNA was quantified on Nanodrop 8000 (Thermo Fisher Scientific) and on Qubit 3.0 (Thermo Fisher Scientific). The quality of isolated RNA was analyzed by Fragment Analyzer (Agilent).

### RNA-sequencing library preparation

Total ribo-depleted RNA-seq library preparation was performed as described previously^63^. In brief, 50–500 ng of total RNA were depleted of ribosomal RNA using the Ribo-Zero™ Gold Kit H/M/R Kit (illumina) following manufacturer’s instructions. Ribo-depleted RNA was resuspended in 17 µl of EFP buffer (illumina), heated to 94 °C for 8 min, and used as input for first strand synthesis, using the TruSeq™ RNA Library Preparation Kit v2 (illumina) following manufacturer’s instructions.

### Cell preparation for scRNA-sequencing

According to the experiment, proximal small intestinal crypts or intestinal organoids were resuspended in 1 ml of Single-Cell Isolation Solution (TrypLE supplemented with 1 mg/ml DNase I, 5 mM MgCl_2_, 80 µM Y27632) and incubated for 20 min at 37 °C with short vortexing after first 10 min of incubation. Reaction was quenched by addition of 29 ml ice-cold PBS and cells were centrifuged at 800 × *g* for 5 min at 4 °C. Cell pellet was resuspended in FSM supplemented with 80 µM Y27632. Cells were pre-blocked with the TruStain FcX anti-mouse antibody according to the manufacturer’s specifications. Cells were then treated with CD326 (EpCAM) (G8.8), PE-Cyanine7 coupled rat monoclonal antibody, and different TotalSeq anti-mouse Hashtag antibodies for 30 min on ice in the dark. The cells were then centrifuged at 450 × *g* for 5 min at 4 °C, resuspended in 500 µl fresh FSM and FACS sorted. EpCAM^+^ single cells from young and old mice were flow-sorted into a BSA-coated tube containing 1.5 µl PBS with 0.04% BSA.

*Lamina propria* immune cells were isolated from proximal intestinal tissue. Briefly, after crypt isolation, tissue was chopped and incubated in 3 ml of 1 mg/ml collagenase-D and 1 mg/ml DNase I in RPMI medium supplemented with 2% FBS in an incubator shaker (80 rpm) for 50 min at 37 °C. Tissue was pipetted up and down several times with a p1000 tip. The supernatant was passed through a 100 μm strainer into RPMI medium supplemented with 2% FBS. The remaining tissue was smashed with a syringe plunger and washed with RPMI medium supplemented with 2% FBS to collect the maximum number of cells. The supernatant was centrifuged at 450 × *g*, 4 °C for 5 min. The pellet was resuspended in 40% percoll in RPMI medium supplemented with 2% FBS. The cell suspension was carefully pipetted over 80% percoll in a falcon tube in order to create a gradient. The falcon tubes were centrifuged at 1600 ×*g*, RT for 20 min (centrifuge break disabled). The immune cells were collected carefully from border of the two percoll concentrations and washed with PBS supplemented with 2% FBS. The suspension was centrifuged at 450 × *g*, 4 °C for 5 min. Pellet was resuspended in PBS supplemented with 2% FBS and procced for staining. Cells were blocked with TruStain FcX anti-mouse antibody according to manufacturer’s specifications. Staining and hash-tagging were performed at the same time with FITC-coupled CD45 and different TotalSeq anti-mouse Hashtag antibodies for each compartment. Details on antibodies can be found in Supplementary Table S2.

### Droplet-based scRNA-sequencing

scRNA-seq was performed according to the 10X Genomics protocols. Briefly, the prepared single cell suspension was carefully mixed with reverse transcription mix using the Chromium Single Cell 3’ Library & Gel beads chemistry v2 (10x Genomics) and loaded into a Chromium Single Cell A Chip (10x Genomics).

During the encapsulation process in the 10X Genomics Chromium system, the cells were lysed within the droplet and released polyadenylated RNA, which then bound to the barcoded bead that was captured with the cell. Following the guidelines of the 10x Genomics' user manual, the droplets were directly subjected to reverse transcription, the emulsion was broken and cDNA was purified using Dynabeads MyOne Silane (Thermo Fisher Scientific). After the PCR amplification of cDNA with eight cycles, it underwent purification and a quality control check on the Fragment Analyzer (Agilent).

The cDNA was fragmented for five minutes and dA-tailed, followed by an adapter ligation step and an indexing PCR of 10 cycles in order to generate libraries. After quantification, the libraries were sequenced on NextSeq500 platform (illumina) using a high-output flowcell in PE mode (R1: 26 cycles; I1: 8 cycles; R2: 57 cycles).

### High-throughput sequencing

All the samples for genome-wide experiments were sequenced on the HiSeq2500, and NextSeq500 platform (Illumina, San Diego, CA, USA).

### Quantitative real-time PCR

cDNA synthesis was done with 1 µg of total RNA by using iScript cDNA Synthesis Kit (Biorad) according to the manufacturer’s protocol. Quantitative real-time PCR analysis was performed on Corbett RotorGene 6000 (Qiagen) using SYBR GreenER qPCR SuperMix (Thermo Fisher Scientific). Each reaction was performed in a 19 µl qPCR mix and 1 µl of 1∶10 diluted cDNA. qRT-PCR conditions were 10 min at 95 °C, then 50 cycles of 10 s at 95 °C, 10 s at 56 °C, 20 s at 68 °C and 3 s at 68 °C. To obtain amplicon data, a melting curve analysis was performed after each PCR run: each sample was analyzed in triplicate. The concentrations of samples were calculated using relative standard curve method. All analyzed gene expressions were normalized to housekeeping gene Beta-actin. Primers were designed using the NCBI Primer-BLAST tool and their sequences are listed in Supplementary Table S1.

### Chromatin Immunoprecipitation (ChIP)-qRT-PCR analysis

Chromatin Immunoprecipitation was performed on IFNγ treated and untreated organoids as previously described^63^. Briefly, treated organoids with IFNγ (2 ng/ml) for 24 h were washed, disrupted, and cross-linked by addition of formaldehyde to 1% for 10 min at RT, quenched with 0.125 M glycine for 5 min at RT, and then washed twice with cold PBS. The cross-linked organoids were resuspended in SDS ChIP Buffer (20 mM Tris-HCl pH 8.0, 10 mM EDTA, 1% SDS and protease inhibitors), incubated on a rotator for 30 min at 4 °C, sonicated for 18 cycles on high power setting (30 s ON, 30 s OFF) using the Bioruptor Next Gen (Diagenode) and centrifuged at 12,000 × *g* for 10 min at 4 °C. The isolated chromatin was diluted 10-fold with ChIP dilution buffer (16.7 mM Tris-HCl pH 8.0, 0.01% SDS, 1.1% Triton X-100, 1.2 mM EDTA, 167 mM NaCl) and incubated with 4 µg of antibody overnight at 4 °C on a rotator. Protein G-conjugated magnetic beads (Dynal, Thermo Fisher Scientific) were saturated with PBS/1% BSA and sonicated salmon sperm overnight at 4 °C. Next day, samples were incubated with saturated beads for two hours at 4 °C on a rotator, and subsequently washed with 1 ml of cold Low salt buffer (20 mM Tris-HCl pH 8.0, 0.1 % SDS, 1% Triton X-100, 2 mM EDTA, 150 mM NaCl), 1 ml of cold High salt buffer (20 mM Tris-HCl pH 8.0, 0.1 % SDS, 1% Triton X-100, 2 mM EDTA, 500 mM NaCl), 1 ml of cold LiCl buffer (10 mM Tris-HCl pH 8.0, 1% DOC, 250 mM LiCl, 1 mM EDTA, 1% NP-40), and twice with 1 ml of cold TE buffer (10 mM Tris-HCl pH 8.0, 1 mM EDTA). The immunoprecipitated chromatin was eluted with 200 µl of Elution buffer (10 mM Tris-HCl pH 8.0, 1 mM EDTA, 1% SDS, 150 mM NaCl, 5 mM DTT) for 30 min at RT on a rotator, and decrosslinked at 65 °C overnight. The decrosslinked DNA was purified using QiaQuick PCR Purification Kit (Qiagen) according to the manufacture’s instruction. The immunoprecipitated DNA was analyzed by quantitative real-time PCR using the SYBR GreenERkit (Invitrogen) and the following primers: H2-Ab1 (Forward: CAGGTCCTGACCCCTGTTTA, Reverse: GTTTCAGGAAGGGACAGCCA), Wars (Forward: CTGGCTGTGTAGTCCAAGGG, Reverse: GAAAGGGTGTGGCAAAGCAG). The antibodies used for ChIP were: rabbit anti-Stat1 (9172, Cell Signaling), rabbit anti-IgG (12–370, Millipore). All the antibodies were used at a concentration of 1:250. Details on antibodies can be found in Supplementary Table S2.

### Immunofluorescence on frozen tissue sections

Small piece (2 cm) of proximal intestine (duodenum) was fixed in 4% PFA in PBS overnight at 4 °C on a rotator. After fixation, the tissue was washed three times with PBS for 15 min at RT, and then dehydrated in 20% Sucrose overnight at 4 °C on a rotator. Subsequently, the fixed tissue was mounted in cast using optimal cutting temperature (OCT) compound, slowly frozen using liquid nitrogen, and stored at −80 °C. For immunofluorescence staining, 14 µm sections were cut and the tissue was permeabilized in PBS supplemented with 0.1% Triton X-100 for 10 min and washed three times with PBS for 15 min at RT. The permeabilized tissue was blocked for 1 h with 10% FBS in PBS supplemented with 0.1% Tween 20 (T-PBS), and then incubated with primary antibody in T-PBS supplemented with 2% FBS overnight in a humid chamber at 4 °C. After incubation with primary antibody, the tissue was washed three times with PBS for 15 min at RT, and then incubated with secondary antibody in PBS supplemented with DAPI (1:1000) for 1 h at RT. After incubation with a secondary antibody, the tissue was washed three times with PBS for 15 min at RT and mounted. Imaging was performed at the Fritz Lipmann Institute - Core Facility Imaging. Images were acquired using the Axiovert 200 inverted microscope with ApoTome slider module for optical sectioning. Imaging was performed at 200x total magnification.

Primary antibodies used for immunofluorescence were: rabbit monoclonal anti-EpCAM [EPR20533-63] (Abcam; 1:250); rat monoclonal anti-Mouse MHC Class II (I-A) (NIMR-4), PE (Thermo Fisher Scientific; 1:250). Secondary antibodies used for immunofluorescence were: Alexa Fluor 488 donkey anti-rabbit IgG (H + L) (Thermo Fisher Scientific; 1:500); Alexa Fluor 568 goat anti-rat IgG (H + L) (Thermo Fisher Scientific; 1:500). Details on antibodies can be found in Supplementary Table S2.

### Immunofluorescence on paraffin-embedded tissue sections

5 μm paraffin sections were deparaffinized by three times immersion in xylene (5 min each time) and rehydrated by immersion in a series of graded ethanol dilutions 100%, 90%, and 70% for 5 min each. Epitope retrieval was performed by preheating the sections 5 min at full power microwave (900 W) in 10 mM sodium citrate buffer pH 6.5 until boiling, followed by 10 min at a sub-boiling temperature (600 W). After cooling down for 20 min, the sections were washed in PBS and blocked with 1% BSA/PBS for 1 h at RT in humid chamber. Sections were stained with primary antibodies: anti-Olfm4 (Cell Signaling, D6Y5A, #39141), anti-Muc2 (abcam, ab90007), and anti-Chga (abcam, ab15160) in 1% BSA/PBS for 16 h at 4 degree in humid chamber. This was followed by washing in PBST (0.1% Tween 20, 3 × 5 min) and subsequent incubation for 30 min with secondary anti-rabbit IgG conjugated with AF488. All the antibodies were used at concentration of 1:250. The slides were washed in T-PBS (0.1% Tween 20, 3 × 5 min) and mounted with mounting medium including DAPI. Images of stained sections were acquired using Axio Imager from Zeiss and analyzed by the ZEN blue software v2 (Zeiss). For further image analysis, the graphics tools for counting and measuring the ZEN software were used. Details on antibodies can be found in Supplementary Table S2.

### RNA-sequencing data analysis

Fastq files quality check was performed using FastQC v0.11.5. The fastq files were mapped to the mm9 genome using TopHat v2.1.0 with the following parameters --bowtie1 --no-coverage-search -a 5. The number of reads covered by each gene is calculated by HTSeq-Count 0.11.2 with -s no -a 0 -t exon -m intersection-nonempty parameters. Before further analysis, all the rRNA genes are removed from the count data. For calculating DEGs and normalized count, DESeq2 R package v1.20.0 was used with the default parameters. For Pearson correlation analysis and plotting the expression, the normalized count was used.

### Gene set enrichment analysis

For gene set enrichment analysis, normalized counts (for each gene in all of the samples) were scaled using the scale function in R (with center = TRUE, scale = TRUE parameters). The average of *Z* scores were calculated for each group and used for drawing plots and downstream analysis. The *p* values were calculated using Wilcoxon paired test (two-tailed). Boxplots show the quartile distribution of the data. A distance of 1.5 times the interquartile range (Q3–Q1) is measured out below the lower quartile and a whisker is drawn up to the lower observed point from the dataset that falls within this distance. All other observed points are plotted as outliers.

### scRNA-sequencing data analysis for intestinal crypt cells

The raw sequencing data was processed with the ‘count’ command of the Cell Ranger software (v2.1.0, 10X Genomics) with the default options. The required reference was built with the ‘mkref’ command of Cell Ranger based on the murine genome mm10 as well as the gene annotation from Ensembl (v87) as input. The annotation was filtered with the ‘mkgtf’ command of Cell Ranger to include only protein-coding, lincRNA, and antisense gene features (‘--attribute=gene_biotype:protein_coding --attribute=gene_biotype:lincRNA –attribute=gene_biotype:antisense’). The count file was directly uploaded into R using cellrangerRkit package v2.0.0. Before further analysis on the count data, non-expressed genes were removed and the counts were normalized to the total number of counts in each cell (using normalize_barcode_sums_to_median function in cellrangerRkit package) and transformed to log 10 for all of the downstream analysis. PCA was calculated using prcomp function with center = TRUE,scale. = TRUE parameters. Clustering of the cells was done using kmean clustering (center = 9, nstart = 10) and the cell type of each defined cluster was determined using the well-known markers for each cell types. Using 9 clusters, Tuft and Enteroendocrine cells were in in the same cluster, so we separated these cell types by re-clustering using kmean (center = 2, nstart = 10). To define markers for each cluster we used order_cell_by_clusters and prioritize_top_genes (method = “sseq”, min_mean = 0.1), respectively. The expression of markers was compared between the desired cluster versus all other cells (using prioritize_top_genes function) and the genes with log2 fold change ≥ 2 and adjusted *p* value < 0.05 were selected as the marker.

### scRNA-sequencing data analysis for lamina propria immune cells and intestinal organoid cells

The Cell Ranger Software Suite (Version 3.1.0) was used to perform sample de-multiplexing, barcode processing, and single-cell 3’ UMI counting with mm10-3.0.0 as the reference genome. Effective reads (UMI) per cell were scaled to the same level (median UMI counts per cell: 1330) in each sample by downsampling raw reads. Cells with hashtag reads were defined into different categories of “single hashtag”, “double hashtag”, “triple hashtag” and “multiple hashtag” by the hashtag ratio in each cell. Cells with less than 10 reads mapping to hashtags were discarded with only those defined as “single hashtag” kept for downstream analysis. Afterwards, gene-barcode matrix of all samples was integrated with Seurat v3. Following criteria were then applied to each cell, i.e., for immune cells: gene number between 200 and 1500, UMI counts <5000, and mitochondrial gene normalized counts below 8; for intestinal organoid cells: mitochondrial gene normalized counts below 20. After filtering, a total of remaining 8997 immune cells (4423 cells for young samples, 4574 cells for old samples) and 13,054 intestinal organoid cells (5843 cells for control group, 7211 cells for IFNγ-treated group) were left for following analysis. Batch effect was removed by “canonical correlation analysis (cca)” correction when integrating the single cells from control organoids and IFNγ-treated organoids.

### Dimension reduction, graph clustering, and UMAP/t-SNE visualization

For immune cells, a subset of features (7425 genes) that exhibit high cell-to-cell variation in the dataset is selected with the method of “mvp” in Seurat (with mean cutoff between 0.0125 and 2; dispersion cutoff >0.9), which could identify variable features while controlling for the strong relationship between variability and average expression. Basically, features were divided into 20 bins based on their average expression, and *z* scores were calculated for dispersion within each bin.

For intestinal organoid cells, a subset of features (2000 genes) that exhibit high cell-to-cell variation in the dataset is selected with the method of “vst” in Seurat. The feature values were standardized using the observed mean and expected variance given by the fitted line model of log(variance) and log(mean). Feature variance is then calculated on the standardized values after clipping to a maximum.

Focusing on variable genes in downstream analysis helps to highlight biological signal in single-cell datasets. We conservatively used all the variable genes identified by “mvp” or “vst” method for dimension reduction to ensure that most of the variability in the dataset was maintained. Dimension reduction of the filtered hashtag-isolated gene-barcode matrix was applied by PCA on these variable genes. Then Uniform Manifold Approximation and Projection (UMAP) for intestinal organoids and t-distributed stochastic neighbor embedding (t-SNE) for immune cells was performed on the top 20 principal components for visualizing the cells. Meanwhile, graph-based clustering was executed on the dimension-reduced data with Seurat v3.

### Differential analysis for clusters and identification of cluster-specific genes

Wilcoxon rank sum test, as implemented in Seurat v3 was adopted to achieve differential analysis. For each cluster, DEGs in aging were generated with the average gene expression across all cells in each cluster. In the same manner, the mean expression of each gene from each cluster was compared to that from cells in all other clusters to identify genes that are enriched in a specific cluster. The top few cluster-specific genes in the rank based on their expression difference from each cluster were examined. The centered expression of each gene was used for visualization by heatmap. Classification of immune cell subsets was inferred from the annotation of cluster-specific genes. Different cell types were designated manually by referring to known markers (Supplementary Data S4).

### Testing for shifts in cell proportions in immune cells and intestinal organoids

Odds ratio was calculated to represent the change in relative abundance of each cell type in aging or treatment. Dramatic changes in the frequency of some cell types were observed in these two datasets. The statistical significance of these shifts was assessed by calculating, regarding each condition comparison and cell type, the exact hypergeometric probability (without replacement) of the observed change in cell numbers.

Specifically, given that m and n total cells (of all cell types) are sequenced in a treatment and control condition respectively, we test, for a given cell type, whether the number of k and q of observed cells of type C in total and treatment condition respectively, significantly deviates from a null model given by the hypergeometric distribution. The probability of observing these values was calculated using the R function ‘phyper’ from the ‘stats’ package, using the command: *p* = phyper(q, k, m, n) and was reported as a hypergeometric *p* value. Confidence intervals for the odds ratio were computed using the R function ‘fisher.test’.

### Gene functional annotation

Statistically significant DEGs (adjusted *p* value < 0.05) were uploaded to QIAGEN IPA software for Canonical Pathway and Upstream Regulator analysis (Qiagen 2020, ver. 01-18-06). Enriched pathways and candidate upstream regulators could be retrieved from IPA.

### Motif prediction

Motif prediction was proceeded by software HOMER-v4.9.1 with a setting of “-start −1000 -end 500 -len 8,10 -p 4 -b”. Binomial distribution was used to calculate *p* values in the enriched motifs. Analysis was performed on the promoters of the genes known to be markers of secretory cells in the intestine versus promoters of the genes known markers of enterocytes.

### Statistics and reproducibility

No statistical method was used to predetermine sample size. No data were excluded from the analyses. The Investigators were not blinded to allocation during experiments and outcome assessment.

### Reporting summary

Further information on research design is available in the Nature Portfolio Reporting Summary linked to this article.

### Supplementary information

Supplementary Information

Description of Additional Supplementary Files

Supplementary Data 1

Supplementary Data 2

Supplementary Data 3

Supplementary Data 4

Reporting Summary

### Source data

Source Data

## Data Availability

All raw sequencing data reported in this paper have been deposited on GeoDatasets under the Geo Accession numbers GSE129510, GSE129708, GSE169368, GSE129710, GSE174297, GSE169351. Genome assembly used is mm9. Source data for graphs are provided with this paper. Source data are provided with this paper.

## Code availability

Custom scripts can be found at http://github.com/smmrasa/GSEA.git.
