# Author Correction: IFNγ-Stat1 axis drives aging-associated loss of intestinal tissue homeostasis and regeneration

**DOI:** 10.1038/s41467-023-42168-8

**Published:** 2023-10-09

**Authors:** Omid Omrani, Anna Krepelova, Seyed Mohammad Mahdi Rasa, Dovydas Sirvinskas, Jing Lu, Francesco Annunziata, George Garside, Seerat Bajwa, Susanne Reinhardt, Lisa Adam, Sandra Käppel, Nadia Ducano, Daniela Donna, Alessandro Ori, Salvatore Oliviero, Karl Lenhard Rudolph, Francesco Neri

**Affiliations:** 1 Leibniz Institute on Aging – Fritz Lipmann Institute (FLI), Jena, Germany; 2 Department of Life Sciences and Systems Biology, University of Turin, Torino, Italy; 3 Molecular Biotechnology Center, University of Turin, Torino, Italy; 4 Dresden-concept Genome Center, c/o Center for Regenerative Therapies Dresden (CRTD), Dresden, Germany

**Keywords:** Endoderm, Gastrointestinal models, Intestinal stem cells

Correction to: *Nature Communications* 10.1038/s41467-023-41683-y, published online 30 September 2023

In this article the author name Dovydas Sirvinskas was incorrectly written as Dovydas Syrvinskas. The original article has been corrected.

