# Supplementary material for: IFNγ-Stat1 axis drives aging-associated loss of intestinal tissue homeostasis and regeneration
Source: Nat Commun. 2023 Sep 30;14:6109. doi: 10.1038/s41467-023-41683-y (PMC10542816; doi:10.1038/s41467-023-41683-y)
Supplement: Supplementary file 1 — Supplementary Information [file 41467_2023_41683_MOESM1_ESM.pdf]

Title

IFNγ-Stat1 axis drives aging-associated loss of intestinal tissue homeostasis and regeneration

SUPPLEMENTARY INFORMATION

Supplementary information contains 7 Supplementary Figures, 2 Supplementary Tables and 4 Supplementary Data files.

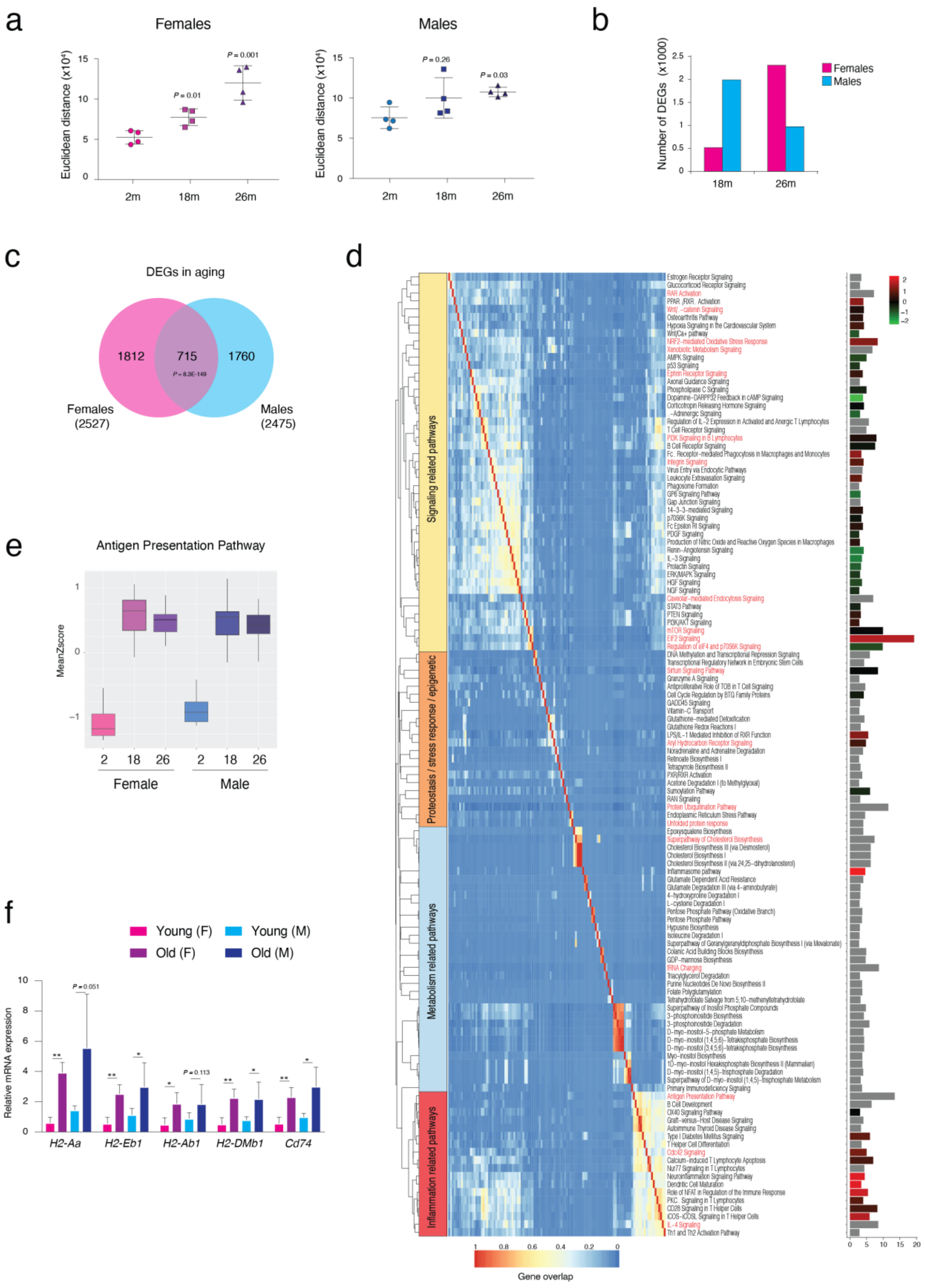
a
Females
Males
Euclidean distance (x10⁴)
P = 0.01
P = 0.001
P = 0.26
P = 0.03
2m
18m
26m
b
Number of DEGs (x1000)
Females
Males
18m
26m
c
DEGs in aging
1812
715
1760
P = 8.3E-149
Females (2527)
Males (2475)
d
Signaling related pathways
Proteostasis / stress response / epigenetic
Metabolism related pathways
Inflammation related pathways
Estrogen Receptor Signaling
Glucocorticoid Receptor Signaling
RAR Activation
PPAR /RXR Activation
Wnt/ -catenin Signaling
Osteoarthritis Pathway
Hypoxia Signaling in the Cardiovascular System
Wnt/Ca+ pathway
NRF2-mediated Oxidative Stress Response
Xenobiotic Metabolism Signaling
AMPK Signaling
p53 Signaling
Ephrin Receptor Signaling
Axonal Guidance Signaling
Phospholipase C Signaling
Dopamine-DARPP32 Feedback in cAMP Signaling
Corticotropin Releasing Hormone Signaling
-Adrenergic Signaling
Regulation of IL-2 Expression in Activated and Anergic T Lymphocytes
T Cell Receptor Signaling
PI3K Signaling in B Lymphocytes
B Cell Receptor Signaling
Fc Receptor-mediated Phagocytosis in Macrophages and Monocytes
Integrin Signaling
Virus Entry via Endocytic Pathways
Leukocyte Extravasation Signaling
Phagosome Formation
GP6 Signaling Pathway
Gap Junction Signaling
14-3-3-mediated Signaling
p70S6K Signaling
Fc Epsilon RI Signaling
PDGF Signaling
Production of Nitric Oxide and Reactive Oxygen Species in Macrophages
Renin-Angiotensin Signaling
IL-3 Signaling
Prolactin Signaling
ERK/MAPK Signaling
HGF Signaling
NGF Signaling
Caveolar-mediated Endocytosis Signaling
STAT3 Pathway
PTEN Signaling
PI3K/AKT Signaling
mTOR Signaling
EIF2 Signaling
Regulation of eIF4 and p70S6K Signaling
DNA Methylation and Transcriptional Repression Signaling
Transcriptional Regulatory Network in Embryonic Stem Cells
Sirtuin Signaling Pathway
Granzyme A Signaling
Antiproliferative Role of TOB in T Cell Signaling
Cell Cycle Regulation by BTG Family Proteins
GADD45 Signaling
Vitamin-C Transport
Glutathione-mediated Detoxification
Glutathione Redox Reactions I
LPS/IL-1 Mediated Inhibition of RXR Function
Aryl Hydrocarbon Receptor Signaling
Noradrenaline and Adrenaline Degradation
Retinoate Biosynthesis I
Tetrapyrrole Biosynthesis II
PXR/RXR Activation
Acetone Degradation I (to Methylglyoxal)
Sumoylation Pathway
RAN Signaling
Protein Ubiquitination Pathway
Endoplasmic Reticulum Stress Pathway
Unfolded protein response
Epoxysqualene Biosynthesis
Superpathway of Cholesterol Biosynthesis
Cholesterol Biosynthesis III (via Desmosterol)
Cholesterol Biosynthesis I
Cholesterol Biosynthesis II (via 24,25-dihydrolanosterol)
Inflammasome pathway
Glutamate Dependent Acid Resistance
Glutamate Degradation III (via 4-aminobutyrate)
4-hydroxyproline Degradation I
L-cysteine Degradation I
Pentose Phosphate Pathway (Oxidative Branch)
Pentose Phosphate Pathway
Hypusine Biosynthesis
Isoleucine Degradation I
Superpathway of Geranylgeranyldiphosphate Biosynthesis I (via Mevalonate)
Colanic Acid Building Blocks Biosynthesis
GDP-mannose Biosynthesis
tRNA Charging
Triacylglycerol Degradation
Purine Nucleotides De Novo Biosynthesis II
Folate Polyglutamylation
Tetrahydrofolate Salvage from 5,10-methenyltetrahydrofolate
Superpathway of Inositol Phosphate Compounds
3-phosphoinositide Biosynthesis
3-phosphoinositide Degradation
D-myo-inositol-5-phosphate Metabolism
D-myo-inositol (1,4,5,6)-Tetrakisphosphate Biosynthesis
D-myo-inositol (3,4,5,6)-tetrakisphosphate Biosynthesis
Myo-inositol Biosynthesis
1D-myo-inositol Hexakisphosphate Biosynthesis II (Mammalian)
D-myo-inositol (1,4,5)-trisphosphate Degradation
Superpathway of D-myo-inositol (1,4,5)-trisphosphate Metabolism
Primary Immunodeficiency Signaling
Antigen Presentation Pathway
B Cell Development
OX40 Signaling Pathway
Graft-versus-Host Disease Signaling
Autoimmune Thyroid Disease Signaling
Type I Diabetes Mellitus Signaling
T Helper Cell Differentiation
Cdc42 Signaling
Calcium-induced T Lymphocyte Apoptosis
Nur77 Signaling in T Lymphocytes
Neuroinflammation Signaling Pathway
Dendritic Cell Maturation
Role of NFAT in Regulation of the Immune Response
PKC Signaling in T Lymphocytes
CD28 Signaling in T Helper Cells
iCOS-iCOSL Signaling in T Helper Cells
IL-4 Signaling
Th1 and Th2 Activation Pathway
Gene overlap
e
Antigen Presentation Pathway
MeanZscore
2
18
26
Female
Male
f
Young (F)
Young (M)
Old (F)
Old (M)
Relative mRNA expression
P = 0.051
P = 0.113
H2-Aa
H2-Eb1
H2-Ab1
H2-DMb1
Cd74

Figure S1. Mouse intestinal crypts show transcriptional alterations during aging.

a) Point plot of the Euclidean sample-to-sample distance in the RNA-seq datasets as in Figure 1a. Error bars represent the SD. *P* value was calculated by t test. m = months. *n* = 4 mice per group were analyzed.

b) Bar chart showing the number of DEGs in aging intestinal crypts measured by RNA-seq as in Figure 1a. m = months.

c) Venn diagram of the DEGs found in the RNA-seq of the intestinal crypts (young versus old animals). *P* value was calculated by hypergeometric distribution test.

d) Left panel: Hierarchical clustering and heatmap of the gene overlap correlation of the top 120 GO pathways found enriched in intestinal crypts during aging. The clustering revealed four main clusters of pathways that can be used for subdividing the pathways according to the main GO category (signaling, metabolism, inflammation and another mixed category composed of stress response, and epigenetics related pathways). Right panel: Bar chart showing the -log10 enrichment p value (x-axis) and activation Z-score (color coded) of the GO pathways.

e) Box plot of the geneset enrichment analysis of the antigen presentation pathway in mouse intestinal crypts from specified ages and in both genders.

f) RT-qPCR validation of the indicated gene expression levels in young (2 months) and old (>20 months) female (F) and male (M) mice. At least *n* = 3 mice per group were analyzed. Error bars represent the SD. *P* value was calculated by Welch's one-tail t test. * = $p < 0.05$; ** = $p < 0.01$.

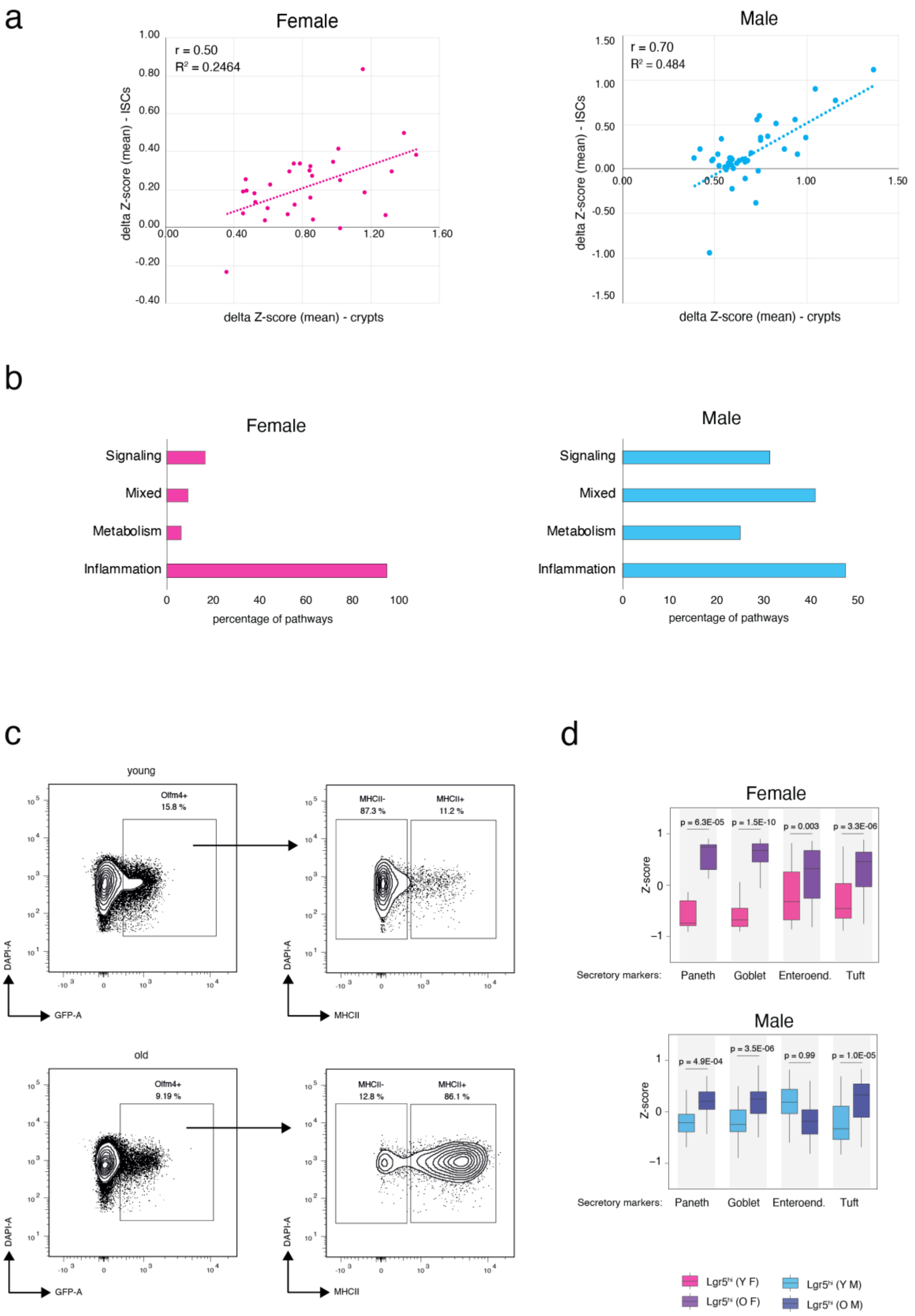
a
Female
r = 0.50
R² = 0.2464
delta Z-score (mean) - ISCs
delta Z-score (mean) - crypts
Male
r = 0.70
R² = 0.484
b
Female
Male
Signaling
Mixed
Metabolism
Inflammation
percentage of pathways
c
young
old
Olfm4+
15.8 %
9.19 %
MHCII-
87.3 %
12.8 %
MHCII+
11.2 %
86.1 %
DAPI-A
GFP-A
MHCII
d
Female
Male
Z-score
p = 6.3E-05
p = 1.5E-10
p = 0.003
p = 3.3E-06
p = 4.9E-04
p = 3.5E-06
p = 0.99
p = 1.0E-05
Secretory markers:
Paneth
Goblet
Enteroend.
Tuft
Lgr5hi (Y F)
Lgr5hi (O F)
Lgr5hi (Y M)
Lgr5hi (O M)

Figure S2. Aged mouse intestinal crypts show gender-specific transcriptional alterations.

a) Scatter plot of delta Z-scores (old minus young Z-scores mean) from the geneset enrichment analysis of the GO pathways found significantly enriched both in the $Lgr5^{hi}$ cells and whole crypt cells. r = Pearson correlation coefficient.

b) Bar chart showing the percentage of GO pathways in each of the indicated main GO categories. Immuno-system and inflammation related pathways are those most commonly regulated between different genders.

c) Representative FACS plots showing percentages of the $MHCII^{+}$ cells within the $Olfm4^{+}$ cells isolated from the intestine of young (upper panel) and old (lower panel) mice.

d) Box plot of geneset enrichment analysis of the marker datasets of the indicated different intestinal secretory cell populations within the $Lgr5^{hi}$ intestinal stem cells purified from female (F) and male (M), young (Y) and old (O) mice. *P* value was calculated by Wilcoxon paired test.

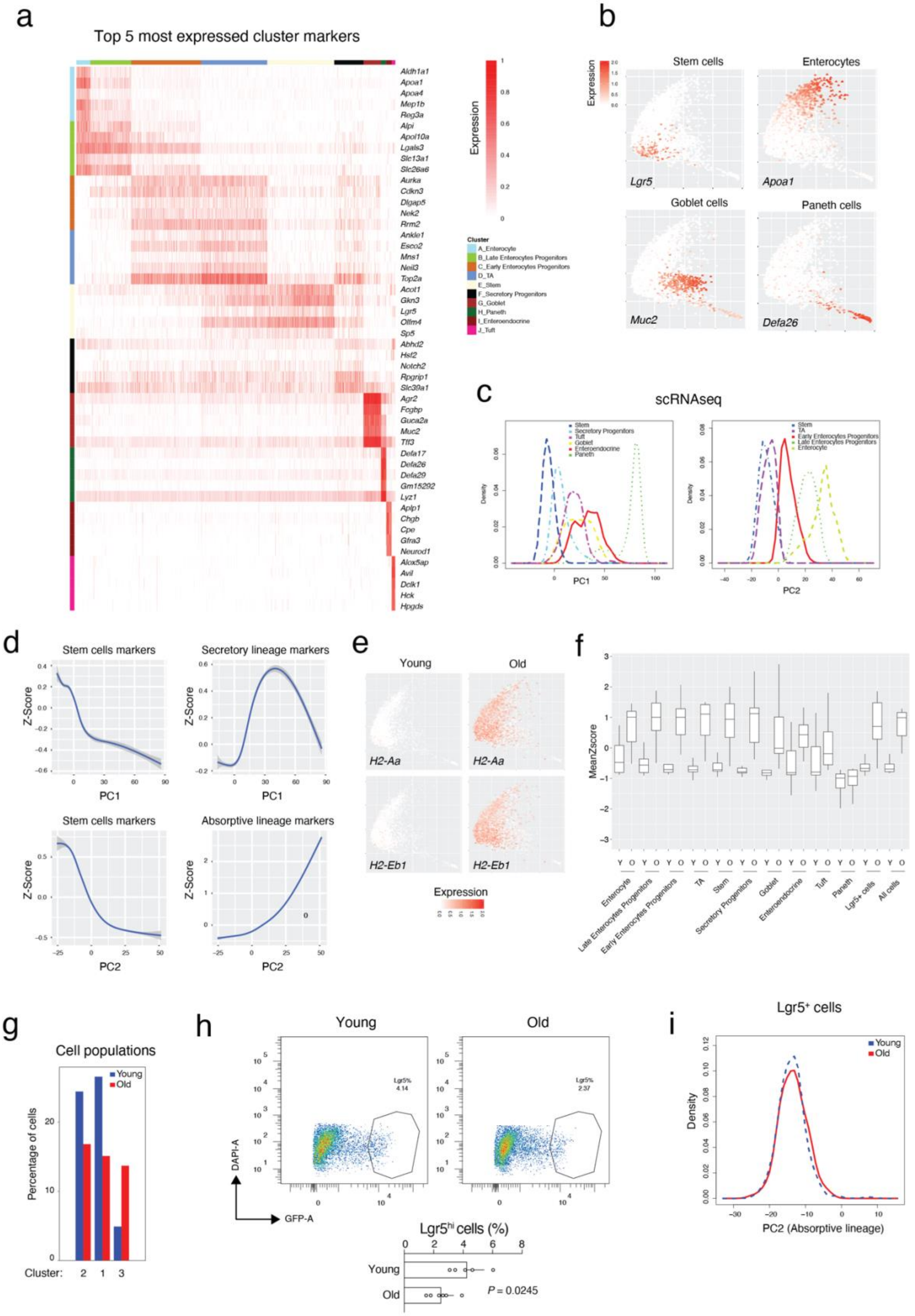

a
Top 5 most expressed cluster markers
Aldh1a1
Apoa1
Apoa4
Mep1b
Reg3a
Alpi
Apol10a
Lgals3
Slc13a1
Slc26a6
Aurka
Cdkn3
Dlgap5
Nek2
Rrm2
Ankle1
Esco2
Mns1
Neil3
Top2a
Acot1
Gkn3
Lgr5
Olfm4
Sp5
Abhd2
Hsf2
Notch2
Rpgrip1
Slc39a1
Agr2
Fcgbp
Guca2a
Muc2
Tff3
Defa17
Defa26
Defa29
Gm15292
Lyz1
Aplp1
Chgb
Cpe
Gfra3
Neurod1
Alox5ap
Avil
Dclk1
Hck
Hpgds
Expression
Cluster
A_Enterocyte
B_Late Enterocytes Progenitors
C_Early Enterocytes Progenitors
D_TA
E_Stem
F_Secretory Progenitors
G_Goblet
H_Paneth
I_Enteroendocrine
J_Tuft
b
Stem cells
Enterocytes
Goblet cells
Paneth cells
Lgr5
Apoa1
Muc2
Defa26
c
scRNAseq
PC1
PC2
Density
d
Stem cells markers
Secretory lineage markers
Absorptive lineage markers
Z-Score
e
Young
Old
H2-Aa
H2-Eb1
f
MeanZscore
Enterocyte
Late Enterocytes Progenitors
Early Enterocytes Progenitors
TA
Stem
Secretory Progenitors
Goblet
Enteroendocrine
Tuft
Paneth
Lgr5+ cells
All cells
g
Cell populations
Percentage of cells
Cluster:
h
DAPI-A
GFP-A
Lgr5%
4.14
2.37
Lgr5hi cells (%)
P = 0.0245
i
Lgr5+ cells
PC2 (Absorptive lineage)

Figure S3. Major Histocompatibility Complex class II (MHCII) is strongly overexpressed during aging in the whole intestinal crypts.

a) Heatmap of the expression of the top 5 markers of each of the 10 clusters identified in the scRNA-seq analysis.

b) Scatter plot of PC1 and PC2 values of the single cell RNA-seq (scRNA-seq) dataset (both the young and the old RNA-seq merged). White-to-red color code represents the expression value of the indicated markers representative of the specified cellular cluster.

c) Line-plot of the cell population density of each of the indicated clusters along the PC1 (upper panel) or PC2 (lower panel) measured by the scRNA-seq experiment.

d) Line-plot of the average Z-score distribution of the indicated geneset markers along the PC1 (left panel) or PC2 (right panel) measured by the scRNA-seq experiment. Z-score is calculated by gene base scaling the count data.

e) Scatter plot of PC1 and PC2 values of the single cell RNA-seq (scRNA-seq) dataset (both the young and the old RNA-seq merged). White-to-red color code represents the expression value of the two indicated markers representative of the antigen presentation pathway.

f) Box plot of the geneset enrichment analysis of the antigen presentation pathway in the specified cell populations from young (Y) or old (O) mouse intestinal crypts.

g) Bar chart indicating the percentage of cells in young or old animals belonging to the indicated clusters in the scRNA-seq. Aging increases the secretory progenitor cluster (cl.3) and reduces stem and TA cells (cl.1-2).

h) Representative FACS plots (upper panels) and relative quantification (bottom panel) showing percentages of the $Lrg5^{+}$ cells in the intestine of mice at the indicated ages. $n \geq 5$ mice per group were analyzed. Error bars represent the SD. *P* value was calculated by Wilcoxon paired test.

i) Line-plot of the cell population density of the Lgr5-expressing cells ($Lgr5^{+}$) along the PC2 axis measured by the scRNA-seq experiment.

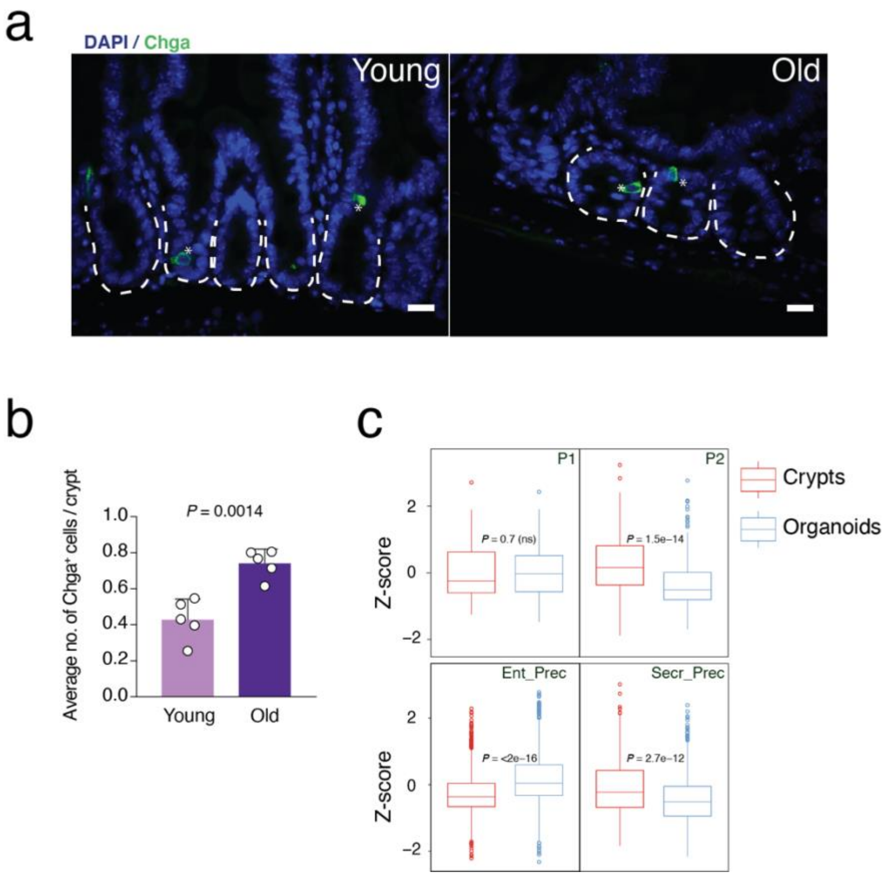

Figure S4. Intestinal cell composition changes during aging.

a) Representative pictures of anti-Chga staining in young and old intestinal crypts. Scale bar, 20 µm. The dotted lines indicate the crypt structure.

b) The bar chart shows the average number of cells positive for Chga in young and old intestinal crypts. *n* = 5 mice per group were analyzed. Error bars represent the SD. *P* value was calculated by Welch's t test.

c) Box plot of geneset enrichment analysis of the indicated gene datasets in freshly isolated intestinal crypts and intestinal organoids. *P* value was calculated by Wilcoxon non-paired test.

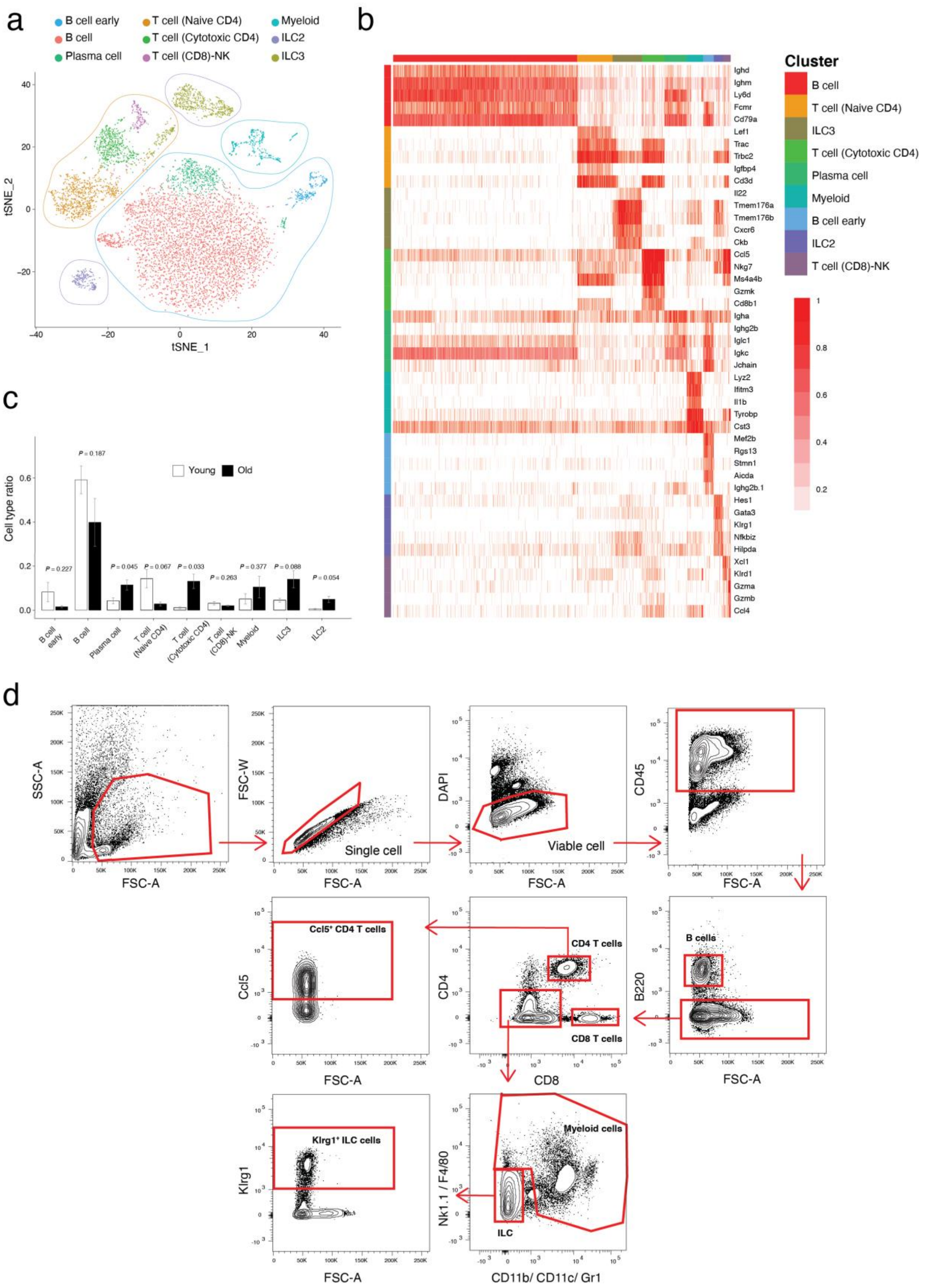

a
B cell early
B cell
Plasma cell
T cell (Naive CD4)
T cell (Cytotoxic CD4)
T cell (CD8)-NK
Myeloid
ILC2
ILC3
tSNE_1
tSNE_2
b
Cluster
B cell
T cell (Naive CD4)
ILC3
T cell (Cytotoxic CD4)
Plasma cell
Myeloid
B cell early
ILC2
T cell (CD8)-NK
Ighd
Ighm
Ly6d
Fcmr
Cd79a
Lef1
Trac
Trbc2
Igfbp4
Cd3d
Il22
Tmem176a
Tmem176b
Cxcr6
Ckb
Ccl5
Nkg7
Ms4a4b
Gzmk
Cd8b1
Igha
Ighg2b
Iglc1
Igkc
Jchain
Lyz2
Ifitm3
Il1b
Tyrobp
Cst3
Mef2b
Rgs13
Stmn1
Aicda
Ighg2b.1
Hes1
Gata3
Klrg1
Nfkbiz
Hilpda
Xcl1
Klrd1
Gzma
Gzmb
Ccl4
c
Young
Old
Cell type ratio
P = 0.227
P = 0.187
P = 0.045
P = 0.067
P = 0.033
P = 0.263
P = 0.377
P = 0.088
P = 0.054
B cell early
B cell
Plasma cell
T cell (Naive CD4)
T cell (Cytotoxic CD4)
T cell (CD8)-NK
Myeloid
ILC3
ILC2
d
SSC-A
FSC-A
FSC-W
Single cell
DAPI
Viable cell
CD45
B220
B cells
CD4
CD8
CD4 T cells
CD8 T cells
Ccl5
Ccl5+ CD4 T cells
Klrg1
Klrg1+ ILC cells
Nk1.1 / F4/80
CD11b/ CD11c/ Gr1
Myeloid cells
ILC

Figure S5. Aging lamina propria immune cells show compositional changes towards more pro-inflammatory condition.

a) t-SNE projection of all immune cells from young and old mice presenting nine different clusters, identified via shared nearest neighbor modularity optimization-based clustering algorithm, followed by merging of similar clusters.

b) Heatmap of the expression of the top 5 markers of each of the nine clusters identified in the scRNA-seq analysis.

c) Bar chart indicating the percentage of cells in young or old animals belonging to the indicated clusters in the scRNA-seq. Error bars represent the SE. *P* value was calculated by non-paired t test.

d) Gating strategy of FACS analysis for immune cell populations in freshly isolated cells from intestinal lamina propria.

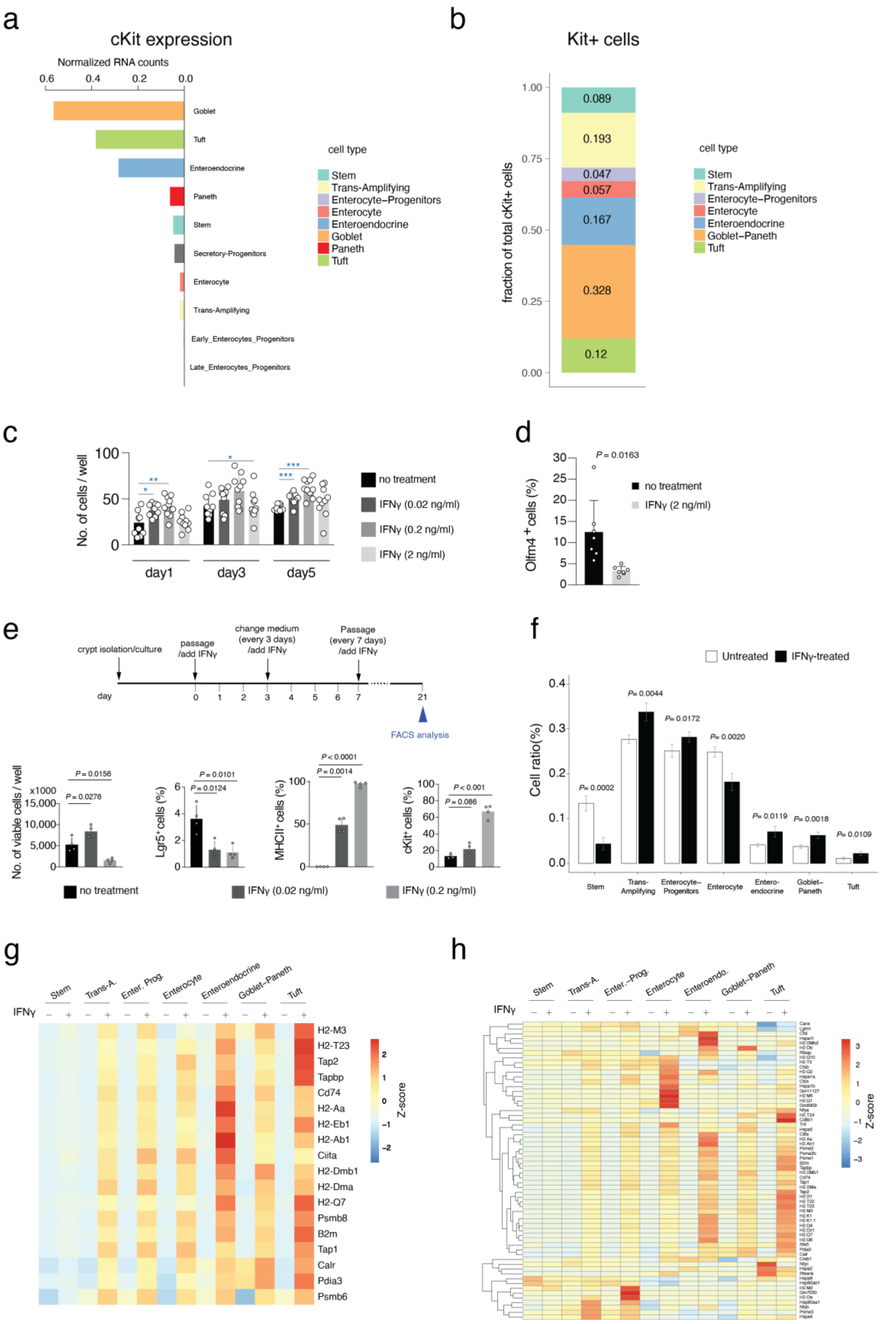

a
cKit expression
Normalized RNA counts
0.6
0.4
0.2
0.0
Goblet
Tuft
Enteroendocrine
Paneth
Stem
Secretory-Progenitors
Enterocyte
Trans-Amplifying
Early_Enterocytes_Progenitors
Late_Enterocytes_Progenitors
cell type
Stem
Trans-Amplifying
Enterocyte−Progenitors
Enterocyte
Enteroendocrine
Goblet
Paneth
Tuft
b
Kit+ cells
fraction of total cKit+ cells
1.00
0.75
0.50
0.25
0.00
0.089
0.193
0.047
0.057
0.167
0.328
0.12
cell type
Stem
Trans-Amplifying
Enterocyte−Progenitors
Enterocyte
Enteroendocrine
Goblet−Paneth
Tuft
c
No. of cells / well
100
50
0
day1
day3
day5
no treatment
IFNγ (0.02 ng/ml)
IFNγ (0.2 ng/ml)
IFNγ (2 ng/ml)
d
Olfm4+ cells (%)
P = 0.0163
no treatment
IFNγ (2 ng/ml)
e
crypt isolation/culture
passage /add IFNγ
change medium (every 3 days) /add IFNγ
Passage (every 7 days) /add IFNγ
day
FACS analysis
No. of viable cells / well
x1000
P = 0.0156
P = 0.0276
Lgr5+ cells (%)
P = 0.0101
P = 0.0124
MHCII+ cells (%)
P < 0.0001
P = 0.0014
cKit+ cells (%)
P < 0.001
P = 0.086
no treatment
IFNγ (0.02 ng/ml)
IFNγ (0.2 ng/ml)
f
Untreated
IFNγ-treated
Cell ratio(%)
P= 0.0002
P= 0.0044
P= 0.0172
P= 0.0020
P= 0.0119
P= 0.0018
P= 0.0109
Stem
Trans-Amplifying
Enterocyte–Progenitors
Enterocyte
Entero-endocrine
Goblet–Paneth
Tuft
g
Stem
Trans-A.
Enter. Prog.
Enterocyte
Enteroendocrine
Goblet–Paneth
Tuft
IFNγ
H2-M3
H2-T23
Tap2
Tapbp
Cd74
H2-Aa
H2-Eb1
H2-Ab1
Ciita
H2-Dmb1
H2-Dma
H2-Q7
Psmb8
B2m
Tap1
Calr
Pdia3
Psmb6
Z-score
h
Stem
Trans-A.
Enter.-Prog.
Enterocyte
Enteroendo.
Goblet–Paneth
Tuft
IFNγ
Z-score

Figure S6. *In vitro* treatment of intestinal organoids with IFNγ could mimic aging induced changes in intestinal crypt cells.

a) Normalized RNA counts for Kit gene in different clusters of scRNA-seq from intestinal crypt cells.

b) Percentage (y axis) of $cKit^+$ cells within each identified cluster (color legend).

c) Similar number of organoids were treated with different concentrations of IFNγ and after 5 days absolute cell number was quantified for each well. Each dot represents one mouse.

d) The bar chart shows the percentage of $Olfm4^+$ cells from intestinal organoids treated with IFNγ after 24h. *n* = 7 mice per group were analyzed. Error bars represent the SD. *P* value was calculated by Welch's t test.

e) Experimental scheme of long-term IFNγ treatment of intestinal organoids and FACS analysis of organoids stimulated with two concentrations of IFNγ for 21 days to assess viable cells, $Lgr5^+$ cells, $MHCII^+$ cells and $cKit^+$ cells. *n* = 4 mice per group were analyzed. Error bars represent the SD. *P* value was calculated by Welch's t test.

f) Bar chart indicating the percentage of cells belonging to the indicated clusters in the scRNA-seq from organoids after IFNγ treatment for 24 hours with 2ng/ml dose. Error bars represent the SD. *P* value was calculated by non-paired t test.

g) Heatmap of the expression level (scaled by gene in the row) of the MHCII genes in the different clusters found in the scRNA-seq of *in vitro* treated intestinal organoids with 2 ng/ml IFNγ for 24h.

h) Hierarchical clustering and heatmap of the expression level of all the genes in the whole Antigen Presentation pathway in the different clusters found in the scRNA-seq of *in vitro* treated intestinal organoids with 2 ng/ml IFNγ for 24h.

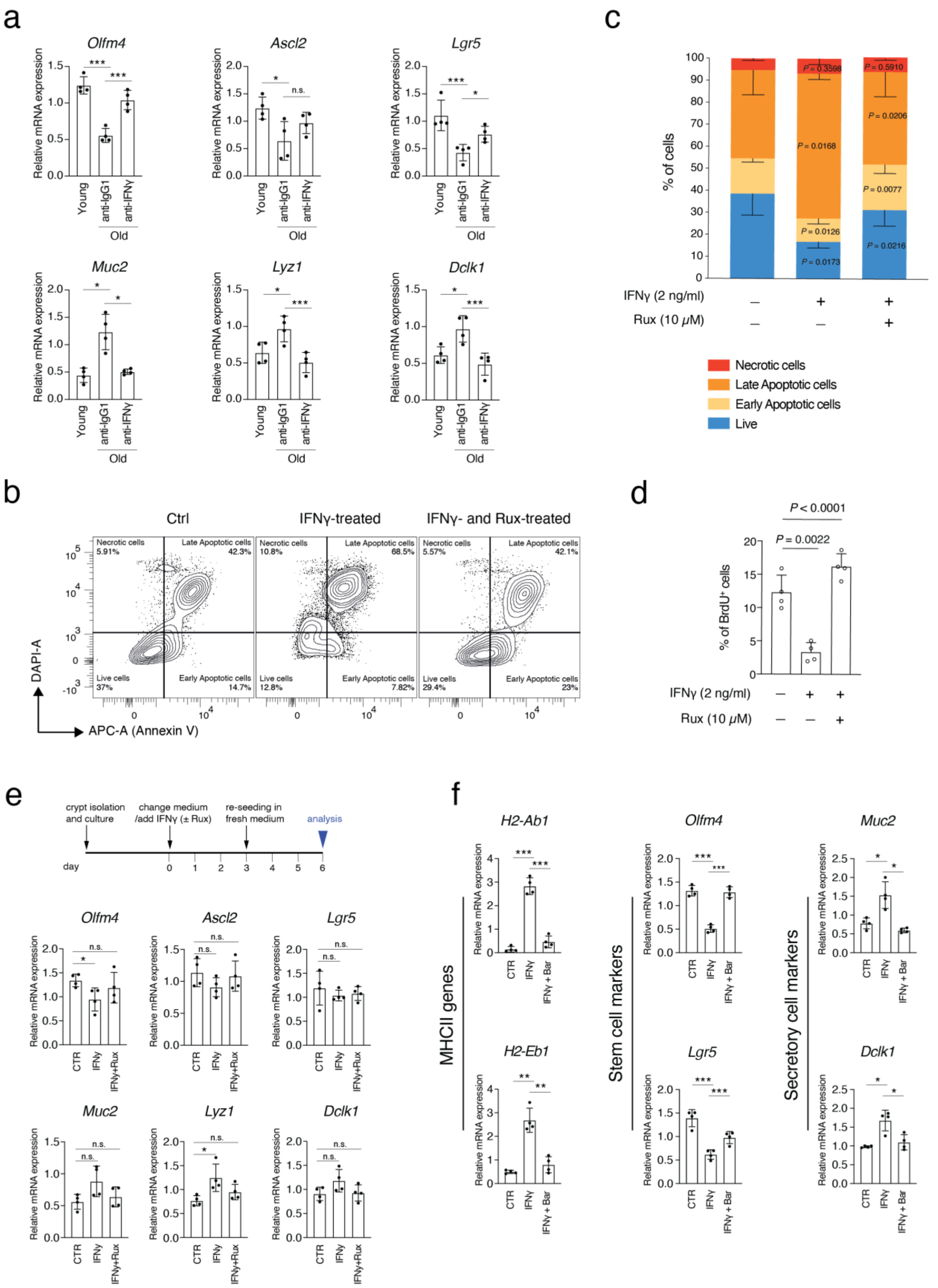

a
Olfm4
Ascl2
Lgr5
Muc2
Lyz1
Dclk1
Relative mRNA expression
Young
anti-IgG1
anti-IFNγ
Old
n.s.
c
% of cells
P = 0.3598
P = 0.5910
P = 0.0206
P = 0.0168
P = 0.0077
P = 0.0126
P = 0.0216
P = 0.0173
IFNγ (2 ng/ml)
Rux (10 μM)
Necrotic cells
Late Apoptotic cells
Early Apoptotic cells
Live
b
Ctrl
IFNγ-treated
IFNγ- and Rux-treated
Necrotic cells 5.91%
Late Apoptotic cells 42.3%
Live cells 37%
Early Apoptotic cells 14.7%
Necrotic cells 10.8%
Late Apoptotic cells 68.5%
Live cells 12.8%
Early Apoptotic cells 7.82%
Necrotic cells 5.57%
Late Apoptotic cells 42.1%
Live cells 29.4%
Early Apoptotic cells 23%
DAPI-A
APC-A (Annexin V)
d
P < 0.0001
P = 0.0022
% of BrdU+ cells
IFNγ (2 ng/ml)
Rux (10 μM)
e
crypt isolation and culture
change medium /add IFNγ (± Rux)
re-seeding in fresh medium
analysis
day
Olfm4
Ascl2
Lgr5
Muc2
Lyz1
Dclk1
Relative mRNA expression
CTR
IFNγ
IFNγ+Rux
n.s.
f
H2-Ab1
H2-Eb1
MHCII genes
Olfm4
Lgr5
Stem cell markers
Muc2
Dclk1
Secretory cell markers
Relative mRNA expression
CTR
IFNγ
IFNγ + Bar

Figure S7. IFNγ regulates intestinal homeostasis.

a) qRT-PCR analysis of stem cell markers (Olfm4, Ascl2 and Lgr5) and of secretory cell markers (Muc2, Lyz1, and Dclk1) in intestinal crypts of mice of the indicated conditions. n = 4 mice per group were analyzed. Error bars represent the SD. P value was calculated by Welch's t test.

b,c) FACS apoptosis assay using Annexin V staining of cells from organoids treated as indicated. b) representative FACS profile. c) quantification of cells in the 4 gates. n = 4 mice per group were analyzed. Organoids treated with IFNγ (2 ng/ml) with or without Ruxolitinib (Rux) for 3 days. Error bars represent the SD. P value was calculated by Welch's t test.

d) FACS analysis of $BrdU^+$ cells in the indicated conditions. n = 4 mice per group were analyzed. Organoids treated with IFNγ (2 ng/ml) with or without Ruxolitinib (Rux) for 3 days. Error bars represent the SD. P value was calculated by Welch's t test.

e) qRT-PCR analysis of stem cell markers (Olfm4, Ascl2 and Lgr5) and of secretory cell markers (Muc2, Lyz1, and Dclk1) in intestinal organoids treated as indicated. IFNγ used at 0.2ng/ml and Ruxolitinib at 10 µM. n = 4 mice per group were analyzed. Error bars represent the SD. P value was calculated by Welch's t test.

f) qRT-PCR analysis of MHCII genes, stem and secretory cell marker genes expression in intestinal organoids treated as indicated. IFNγ used at 0.2 ng/ml and Baricitinib at 2 µM. n = 4 mice per group were analyzed. Error bars represent the SD. P value was calculated by Welch's t test.

Supplementary Tables:

Table S1. Primers for for Real-time qPCR. Related to Methods.

Primer	Sequence
beta Actin_F	TCTTTGCAGCTCCTTCGTTG
beta Actin_R	ACGATGGAGGGGAATACAGC
H2-Aa_F	AGCCCAACACCCTCATCTGC
H2-Aa_R	ACCGTCTGCGACTGACTTGC
H2-Eb1-F	GCGGAGAGTTGAGCCTACG
H2-Eb1-R	ACCATCTGACTTCAATGTTGCC
H2-Ab1-F	GAGATCCTGGAGCGAACG
H2-Ab1-R	AGGGAGATGACGACATTGG
H2-DMb_F	GTCCTCAGTCTGCACTGTATG
H2-DMb_R	CAGCACCCCAAATTCACAG
CD74_F	GCTCCACCTAAAGAGCCACT
CD74_R	GGGTGACTTGACCCAGTTCC
Olfm4_F	CAGCCACTTTCCAATTTCACTG
Olfm4_R	GCTGGACATACTCCTTCACCTTA
Ascl2_F	AAGCACACCTTGACTGGTACG
Ascl2_R	AAGTGGACGTTTGCACCTTCA
Lgr5_F	CAGGTCAATACCGGAGCGAG
Lgr5_R	GCGAGGCACCATTCAAAGTC
Chga_F	AGAGCAGAGGACCAGGAGCTA
Chga_R	TGAGGGGCAAAGGGGGTAAC
Lyz1_F	ATCGTTGTGAGTTGGCCAGAA
Lyz1_R	AGCAGAGCACTGCAATTGATCC
Muc2_F	TGGATCCTCCACGGCAATCC
Muc2_R	GCTGCCCTGCACTCAATGTC

Dclk1_F	CTGGTAACGGAACTTCTCTGG
Dclk1_R	GTACACTCTGGATGGGAAGCA
Stat1_F	TCACAGTGGTTCGAGCTTCAG
Stat1_R	GCAAACGAGACATCATAGGCA
Wars_F	AAAGCCAGCGAGGATTTTGTG
Wars_R	AGCTCTTTGTCAATTTTGCTGC

Table S2. Table of Material used. Related to Methods and Materials.

REAGENT or RESOURCE	SOURCE	IDENTIFIER
Rabbit monoclonal anti-EpCAM (EPR20533-63)	Abcam	Cat# ab221552
CD326 (EpCAM) Monoclonal Antibody (G8.8), PE-Cyanine7, eBioscience™	Thermo Fisher Scientific	Cat# 25-5791-80; RRID:AB_1724047
Stat1 Antibody	Cell Signaling Technology	Cat# 9172, RRID:AB_2198300
Rabbit Normal igg Control antibody, Unconjugated	Millipore	Cat# 12-370, RRID:AB_145841
Rat monoclonal anti-Mouse MHC Class II (I-A) (NIMR-4), PE	Thermo Fisher Scientific	Cat# 12-5322-81; RRID:AB_465930
Alexa Fluor 488 donkey anti-rabbit IgG (H+L)	Thermo Fisher Scientific	Cat# A21206; RRID:AB_2535792
Alexa Fluor 568 goat anti-rat IgG (H+L)	Thermo Fisher Scientific	Cat# A11077; RRID:AB_2534121
Olfm4 (D6Y5A) XP® Rabbit mAb (Mouse Specific) #39141 antibody	Cell Signaling Technology	Cat# 39141, RRID:AB_2650511
MUC2 antibody	Abcam	Cat# ab90007, RRID:AB_10713220
Rabbit Anti-Chromogranin A Polyclonal Antibody, Unconjugated	Abcam	Cat# ab15160, RRID:AB_301704
TruStain FcX(TM) (anti-mouse CD16/32) antibody	BioLegend	Cat# 101319, RRID:AB_1574973
FITC anti-mouse CD45 antibody	BioLegend	Cat# 103108, RRID:AB_312973
TotalSeq(TM)-A0301 anti-mouse Hashtag 1 antibody	BioLegend	Cat# 155801, RRID:AB_2750032
TotalSeq(TM)-A0302 anti-mouse Hashtag 2 antibody	BioLegend	Cat# 155803, RRID:AB_2750033
TotalSeq(TM)-A0303 anti-mouse Hashtag 3 antibody	BioLegend	Cat# 155805, RRID:AB_2750034

TotalSeq(TM)-A0304 anti-mouse Hashtag 4 antibody	BioLegend	Cat# 155807, RRID:AB_2750035
TotalSeq(TM)-A0305 anti-mouse Hashtag 5 antibody	BioLegend	Cat# 155809, RRID:AB_2750036
TotalSeq(TM)-A0306 anti-mouse Hashtag 6 antibody	BioLegend	Cat# 155811, RRID:AB_2750037
TotalSeq(TM)-A0307 anti-mouse Hashtag 7 antibody	BioLegend	Cat# 155813, RRID:AB_2750039
TotalSeq(TM)-A0308 anti-mouse Hashtag 8 antibody	BioLegend	Cat# 155815, RRID:AB_2750040
TotalSeq(TM)-A0309 anti-mouse Hashtag 9 antibody	BioLegend	Cat# 155817, RRID:AB_2750042
TotalSeq(TM)-A0310 anti-mouse Hashtag 10 antibody	BioLegend	Cat# 155819, RRID:AB_2750043
TotalSeq(TM)-A0311 anti-mouse Hashtag 11 antibody	BioLegend	Cat# 155821, RRID:AB_2750136
TotalSeq(TM)-A0312 anti-mouse Hashtag 12 antibody	BioLegend	Cat# 155823, RRID:AB_2750137
InVivoMab anti-mouse IFNγ antibody	Bio X Cell	Cat# BE0055, RRID:AB_1107694
InVivoMab rat IgG1 isotype control (anti-HRP) antibody	Bio X Cell	Cat# BE0088, RRID:AB_1107775
Qiazol Lysis Reagent	Qiagen	Cat# 79306
TrypLE Express Enzyme	Thermo Fisher Scientific	Cat# 12604021
OCT Q PATH® Mounting Media for Cryotomy	VWR	Cat# 00411243
DNase I grade II, f. bov. pancreas	Sigma-Aldrich	Cat# 10104159001
Y27632	Abcam	Cat# ab120129
DAPI	Sigma-Aldrich	Cat# D9542
Matrigel® Growth Factor Reduced (GFR) Basement Membrane Matrix	Corning	Cat# 35623
Recombinant Murine EGF	Preprotech	Cat# 315-09
Recombinant Murine Noggin	Preprotech	Cat# 250-38
Recombinant Human R-Spondin-1	Preprotech	Cat# 120-38
Recombinant Murine IFN-γ	Preprotech	Cat# 315-05
Percoll	Sigma-Aldrich	Cat# P1644
Ruxolitinib (INCB018424)	Selleckchem	Cat# S1378
Baricitinib (LY3009104, INCB028050)	Selleckchem	Cat# S2851
Collagenase-D	Merck	Cat# 11088866001
Sonicated Salmon Sperm DNA	Agilent Technologies	Cat# 201190
ZR-Duet™ DNA/RNA MiniPrep Plus Kit	Zymo Research	Cat# D7003
Ribo-Zero™ Gold Kit H/M/R Kit	illumina	Cat# MRZG12324
TruSeq™ RNA Library Preparation Kit v2	illumina	Cat# RS-122-2001
Chromium™ Single Cell 3' Library & Gel Bead Kit v2	10X Genomics	Cat# PN-120237
Chromium™ Single Cell A Chip Kit	10X Genomics	Cat# PN-120236
Dynabeads™ MyOne™ Silane	Thermo Fisher Scientific	Cat# 37002D
iScript™ cDNA Synthesis Kit	Bio-Rad Laboratories	Cat# 1708891

SYBR GreenER™ qPCR SuperMix for iCycler® Instrument	Thermo Fisher Scientific	Cat# 11761500
QIAquick PCR Purification Kit	Qiagen	Cat# 2810
Qubit® dsDNA HS Assay Kit	Thermo Fisher Scientific	Cat# Q32854
Qubit® RNA HS Assay Kit	Thermo Fisher Scientific	Cat# Q32855
Standard Sensitivity RNA Analysis Kit (15nt)	Agilent	Cat# DNF-471-0500
High Sensitivity RNA Analysis Kit (15nt)	Agilent	Cat# DNF-472-0500
Dynabeads™ Protein G for Immunoprecipitation	Thermo Fisher Scientific	Cat# 10003D
Agencourt AMPure XP	Beckman Coulter	Cat# A63881
Apoptosis detection Kit	BD Bioscience	Cat# 556547
BrdU flow kit	BD Bioscience	Cat# 559619
