# Supplementary material for: IFNγ-Stat1 axis drives aging-associated loss of intestinal tissue homeostasis and regeneration
Source: Nat Commun. 2023 Sep 30;14:6109. doi: 10.1038/s41467-023-41683-y (PMC10542816; doi:10.1038/s41467-023-41683-y)
Supplement: Supplementary file 2 — Description of Additional Supplementary Files [file 41467_2023_41683_MOESM2_ESM.pdf]

Description of Additional Supplementary Files

Supplementary Data 1. Aged mouse intestinal crypts show gender-specific transcriptional alterations. Related to Figure 1.

Differentially expressed gene list of total RNA sequencing from crypt and Lgr5 cells (male and female). All of the old samples are compared to the young age group (2 months old).

Supplementary Data 2. Aged mouse intestinal crypts show gender-specific transcriptional alterations. Related to Figure 1.

Canonical pathway analysis of the deferentially expressed genes (Supplementary Data S1) in male and female crypts.

Supplementary Data 3. Aged intestinal stem cells show specific signaling alterations and an increase of a transcriptionally-primed secretory precursor population. Related to Figure 1,2.

Significant markers for each cell population in single cell RNA sequencing. The markers with adjusted p-value less than 0.05 and log2 fold change of expression more than 1 are selected.

Supplementary Data 4. Aged intestinal lamina propria immune cells show alteration in cell composition. Related to Figure 4, S5.

Identification markers for each subset of lamina propria immune cells are summarized.
