# Supplementary material for: IFNγ-Stat1 axis drives aging-associated loss of intestinal tissue homeostasis and regeneration
Source: Nat Commun. 2023 Sep 30;14:6109. doi: 10.1038/s41467-023-41683-y (PMC10542816; doi:10.1038/s41467-023-41683-y)
Supplement: Supplementary file 7 — Reporting Summary [file 41467_2023_41683_MOESM7_ESM.pdf]

nature portfolio

Corresponding author(s): Francesco Neri

Last updated by author(s): Sep 7, 2023

Reporting Summary

Nature Portfolio wishes to improve the reproducibility of the work that we publish. This form provides structure for consistency and transparency in reporting. For further information on Nature Portfolio policies, see our Editorial Policies and the Editorial Policy Checklist.

Statistics

For all statistical analyses, confirm that the following items are present in the figure legend, table legend, main text, or Methods section.

n/a	Confirmed	
☐	☒	The exact sample size (*n*) for each experimental group/condition, given as a discrete number and unit of measurement
☐	☒	A statement on whether measurements were taken from distinct samples or whether the same sample was measured repeatedly
☐	☒	The statistical test(s) used AND whether they are one- or two-sided<br>*Only common tests should be described solely by name; describe more complex techniques in the Methods section.*
☐	☒	A description of all covariates tested
☐	☒	A description of any assumptions or corrections, such as tests of normality and adjustment for multiple comparisons
☐	☒	A full description of the statistical parameters including central tendency (e.g. means) or other basic estimates (e.g. regression coefficient) AND variation (e.g. standard deviation) or associated estimates of uncertainty (e.g. confidence intervals)
☐	☒	For null hypothesis testing, the test statistic (e.g. *F*, *t*, *r*) with confidence intervals, effect sizes, degrees of freedom and *P* value noted<br>*Give P values as exact values whenever suitable.*
☐	☒	For Bayesian analysis, information on the choice of priors and Markov chain Monte Carlo settings
☐	☒	For hierarchical and complex designs, identification of the appropriate level for tests and full reporting of outcomes
☐	☒	Estimates of effect sizes (e.g. Cohen's *d*, Pearson's *r*), indicating how they were calculated

Our web collection on statistics for biologists contains articles on many of the points above.

Software and code

Policy information about availability of computer code

Data collection	No software was used
Data analysis	FastQC v0.11.5 : checking the quality of fastq files<br>TopHat v2.1.0 :mapping the sequenced reads to the mm9 genome<br>HTSeq-Count 0.11.2: Counting the number of reads covering each gene<br>R version 3.6.0<br>DESeq2 R package v1.20.0: Calculating p-value and normalized count for RNA-seq data<br>scran R package v1.12.1: Prediction of cell cycle from RNA-seq data<br>Custom script for gene set enrichment analysis (http://github.com/smmrasa/GSEA.git)<br>DAVID database v6.8: Gene ontology analysis<br>Ingenuity Pathway Analysis (IPA) v45868156: Prediction of potential upstream regulators<br>ImageJ 1.52p: Image analysis<br>Prism 7.0c.:Statistical analysis and plotting

For manuscripts utilizing custom algorithms or software that are central to the research but not yet described in published literature, software must be made available to editors and reviewers. We strongly encourage code deposition in a community repository (e.g. GitHub). See the Nature Portfolio guidelines for submitting code & software for further information.

Data

Policy information about availability of data

All manuscripts must include a data availability statement. This statement should provide the following information, where applicable:

- Accession codes, unique identifiers, or web links for publicly available datasets
- A description of any restrictions on data availability
- For clinical datasets or third party data, please ensure that the statement adheres to our policy

The accession numbers of all raw sequencing data reported in this paper can be found in the table of material used (GSE129510, GSE129708, GSE169368, GSE129710, GSE174297, GSE169351). genome assembly used is mm9. Source data are provided with this paper.

Research involving human participants, their data, or biological material

Policy information about studies with human participants or human data. See also policy information about sex, gender (identity/presentation), and sexual orientation and race, ethnicity and racism.

Reporting on sex and gender	no applicable
Reporting on race, ethnicity, or other socially relevant groupings	no applicable
Population characteristics	no applicable
Recruitment	no applicable
Ethics oversight	no applicable

Note that full information on the approval of the study protocol must also be provided in the manuscript.

Field-specific reporting

Please select the one below that is the best fit for your research. If you are not sure, read the appropriate sections before making your selection.

[x] Life sciences [ ] Behavioural & social sciences [ ] Ecological, evolutionary & environmental sciences

For a reference copy of the document with all sections, see nature.com/documents/nr-reporting-summary-flat.pdf

Life sciences study design

All studies must disclose on these points even when the disclosure is negative.

Sample size	Sample size was not predetermined. The experiments were done with n > 3 biological replicates. Single cell RNAseq for epithelial cells was comparing one pool of young (3 mice) versus one pool of old (3 mice). Sample sizes were selected on reaserch-based common standards and on the minimum number of samples allowing statistic (BMJ 2009;338:a3166).
Data exclusions	No data were excluded from the analysis.
Replication	All the experiments were successfully replicated at least 3 times (except single cell RNAseq from epithelial cells that was done only one but further independently validated by RT-qPCR, immunofluorescence and FACS experiments) also for experiments where only one representative image or result is shown.
Randomization	Biological samples were randomly selected for the experiments. was random
Blinding	No blind experiments were performed.

Reporting for specific materials, systems and methods

We require information from authors about some types of materials, experimental systems and methods used in many studies. Here, indicate whether each material, system or method listed is relevant to your study. If you are not sure if a list item applies to your research, read the appropriate section before selecting a response.

Materials & experimental systems

n/a	Involved in the study
☐	☒ Antibodies
☒	☐ Eukaryotic cell lines
☒	☐ Palaeontology and archaeology
☐	☒ Animals and other organisms
☒	☐ Clinical data
☒	☐ Dual use research of concern
☒	☐ Plants

Methods

n/a	Involved in the study
☒	☐ ChIP-seq
☐	☒ Flow cytometry
☒	☐ MRI-based neuroimaging

Antibodies

Antibodies used

Rabbit monoclonal anti-EpCAM (EPR20533-63) Abcam Cat# ab221552
CD326 (EpCAM) Monoclonal Antibody (G8.8), PE-Cyanine7, eBioscience™ Thermo Fisher Scientific Cat# 25-5791-80; RRID:AB_1724047
Stat1 Antibody Cell Signaling Technology Cat# 9172, RRID:AB_2198300
Rabbit Normal igg Control antibody, Unconjugated Millipore Cat# 12-370, RRID:AB_145841
Rat monoclonal anti-Mouse MHC Class II (I-A) (NIMR-4), PE Thermo Fisher Scientific Cat# 12-5322-81; RRID:AB_465930
Alexa Fluor 488 donkey anti-rabbit IgG (H+L) Thermo Fisher Scientific Cat# A21206; RRID:AB_2535792
Alexa Fluor 568 goat anti-rat IgG (H+L) Thermo Fisher Scientific Cat# A11077; RRID:AB_2534121
Olfm4 (D6Y5A) XP® Rabbit mAb (Mouse Specific) #39141 antibody Cell Signaling Technology Cat# 39141, RRID:AB_2650511
MUC2 antibody Abcam Cat# ab90007, RRID:AB_10713220
Rabbit Anti-Chromogranin A Polyclonal Antibody, Unconjugated Abcam Cat# ab15160, RRID:AB_301704
TruStain FcX(TM) (anti-mouse CD16/32) antibody BioLegend Cat# 101319, RRID:AB_1574973
FITC anti-mouse CD45 antibody BioLegend Cat# 103108, RRID:AB_312973
TotalSeq(TM)-A0301 anti-mouse Hashtag 1 antibody BioLegend Cat# 155801, RRID:AB_2750032
TotalSeq(TM)-A0302 anti-mouse Hashtag 2 antibody BioLegend Cat# 155803, RRID:AB_2750033
TotalSeq(TM)-A0303 anti-mouse Hashtag 3 antibody BioLegend Cat# 155805, RRID:AB_2750034
TotalSeq(TM)-A0304 anti-mouse Hashtag 4 antibody BioLegend Cat# 155807, RRID:AB_2750035
TotalSeq(TM)-A0305 anti-mouse Hashtag 5 antibody BioLegend Cat# 155809, RRID:AB_2750036
TotalSeq(TM)-A0306 anti-mouse Hashtag 6 antibody BioLegend Cat# 155811, RRID:AB_2750037
TotalSeq(TM)-A0307 anti-mouse Hashtag 7 antibody BioLegend Cat# 155813, RRID:AB_2750039
TotalSeq(TM)-A0308 anti-mouse Hashtag 8 antibody BioLegend Cat# 155815, RRID:AB_2750040
TotalSeq(TM)-A0309 anti-mouse Hashtag 9 antibody BioLegend Cat# 155817, RRID:AB_2750042
TotalSeq(TM)-A0310 anti-mouse Hashtag 10 antibody BioLegend Cat# 155819, RRID:AB_2750043
TotalSeq(TM)-A0311 anti-mouse Hashtag 11 antibody BioLegend Cat# 155821, RRID:AB_2750136
TotalSeq(TM)-A0312 anti-mouse Hashtag 12 antibody BioLegend Cat# 155823, RRID:AB_2750137
InVivoMab anti-mouse IFNγ antibody Bio X Cell Cat# BE0055, RRID:AB_1107694
InVivoMab rat IgG1 isotype control (anti-HRP) antibody Bio X Cell Cat# BE0088, RRID:AB_1107775
Brilliant Violet 605™ anti-mouse CD45 Antibody BioLegend Cat# 103139
FITC anti-mouse CD3ε Antibody BioLegend Cat# 100305
PE anti-mouse CD4 Antibody BioLegend Cat# 100511
APC/Cy7 anti-mouse/human CD45R/B220 Antibody BioLegend "Cat# 103223 "
PE/Cyanine7 anti-mouse F4/80 Antibody BioLegend Cat# 123113
PerCP/Cyanine5.5 anti-mouse/human CD11b Antibody BioLegend Cat# 101227
APC anti-mouse/human KLRG1 (MAFA) Antibody BioLegend Cat# 138411
APC/Cyanine7 anti-mouse/human KLRG1 (MAFA) Antibody BioLegend Cat# 138425
PE/Cyanine7 anti-mouse NK-1.1 Antibody BioLegend Cat# 108713

Validation

Validation of antibodies was done by the manufacturer.

Animals and other research organisms

Policy information about studies involving animals; ARRIVE guidelines recommended for reporting animal research, and Sex and Gender in Research

Laboratory animals

Transgenic mouse lines:
1) Lgr5-ki-e-GFP-creER
2) Olfm4-ki-e-GFP-creER
Mice used in this study were C57BL6/J, Lgr5-eGFP or Olfm4-eGFP mice that generated in in-house facility. Mice used were both female and male young adult and old (4 to 110 weeks old).

Wild animals	The study does not involve wild animals.
Reporting on sex	Mice used were both female and male young adult and old (4 to 110 weeks old).
Field-collected samples	The study does not involve field-collected samples.
Ethics oversight	Experiments were conducted according to protocols approved by the state government of Thuringia Thüringer Landesamt für Verbraucherschutz (TLV) authority (licenses number: TG/J-0002858/A; TG/J-0003616/A; TG/J-0003681/A; FLI-17-109; FLI-18-005, FLI-20-005).

Note that full information on the approval of the study protocol must also be provided in the manuscript.

Flow Cytometry

Plots

Confirm that:

- [x] The axis labels state the marker and fluorochrome used (e.g. CD4-FITC).
- [x] The axis scales are clearly visible. Include numbers along axes only for bottom left plot of group (a 'group' is an analysis of identical markers).
- [x] All plots are contour plots with outliers or pseudocolor plots.
- [x] A numerical value for number of cells or percentage (with statistics) is provided.

Methodology

Sample preparation	According to the experiment, proximal small intestinal crypts or intestinal organoids were resuspended in 1ml of Single-Cell Isolation Solution (TrypLE supplemented with 1 mg/ml DNaseI, 5 mM MgCl2, 80 µM Y27632) and incubated for 20 min at 37°C with short vortexing after first 10 min of incubation. Reaction was quenched by addition of 29 ml ice-cold PBS and cells were centrifuged at 800g for 5 min at 4°C. Cell pellet was resuspended in FSM supplemented with 80 µM Y27632 and stained with appropriate antibodies.<br>Lamina propria immune cells were isolated from proximal intestinal tissue. Briefly after crypt isolation, tissue was chopped and incubated in 3ml of 1mg/ml collagenase-D and 1mg/ml DNaseI in RPMI medium supplemented with 2% FBS in an incubator shaker (80rpm) for 50min at 37°C. Tissue was pipetted up and down several times with a p1000 tip. The supernatant was passed through strainer into RPMI medium supplemented with 2% FBS. Remaining tissue was smashed with a syringe plunger and washed with RPMI medium supplemented with 2% FBS to collect the maximum number of cells. The supernatant was centrifuged at 450g, 4°C for 5min. The pellet was re-suspended in 40% percoll in RPMI medium supplemented with 2% FBS. The cell suspension was carefully pipetted over 80% percoll in a falcon tube in order to create a gradient. The falcon tubes were centrifuged at 1600g, RT for 20min (centrifuge break disabled). The immune cells were collected carefully from border of the two percoll concentrations and washed with PBS supplemented with 2% FBS. The suspension was centrifuged at 450g, 4°C for 5min. Pellet was resuspended in PBS supplemented with 2% FBS and procced for staining.
Instrument	FACS Aria II, FACS ARIA III (BD Biosciences)
Software	FlowJo 10.7.1. PRISM v.7.
Cell population abundance	Quantification of the cells in the different populations were reported in Figure1G,H, Figure2E, Figure4C-F, Figure5C-F, Figure6G-H, Figure7K-L, FigureS6C-D.
Gating strategy	The FACS gating strategy used in the lamina propria immune cells analysis are described in the FigureS5D of the manuscript.

- [x] Tick this box to confirm that a figure exemplifying the gating strategy is provided in the Supplementary Information.
